# Seizing the silent vision loss: cost–utility analysis of population-based glaucoma screening in India

**DOI:** 10.1136/bmjopen-2024-098113

**Published:** 2025-04-03

**Authors:** Neha Purohit, Sandeep Buttan, Parul Chawla Gupta, Ranjan Kumar Choudhury, Kathirvel Soundappan, Atul Kotwal, Shankar Prinja

**Affiliations:** 1 Department of Community Medicine and School of Public Health, Post Graduate Institute of Medical Education and Research, Chandigarh, India; 2 Sightsavers India, New Delhi, India; 3 Department of Ophthalmology, Post Graduate Institute of Medical Education and Research, Chandigarh, India; 4 National Health Systems Resource Center, New Delhi, India

**Keywords:** Primary Health Care, HEALTH ECONOMICS, Glaucoma, Mass Screening, Health economics, Health Care Costs

## Abstract

**Abstract:**

**Objectives:**

Glaucoma is a major cause of irreversible blindness in India; however, if detected early, its progression can be either prevented or stabilised through appropriate medical or surgical treatment. We aim to evaluate the cost–utility of various models for population-based glaucoma screening at primary health centres in India. We also assess the potential impact of the implementation of a population-based screening programme on overall costs of care for glaucoma.

**Design:**

Cost–utility analysis using a mathematical model comprising a decision tree and Markov model was conducted to simulate relevant costs and health outcomes over a lifetime horizon.

**Setting:**

Screening services were assumed to be delivered at primary health centres in India.

**Participants:**

A hypothetical cohort of different target population groups in terms of age groups and risk of glaucoma (age group 40–75 years, 50–75 years, 40–75 years age group at high risk of glaucoma, 50–75 years age group at high risk of glaucoma) were included in comparative screening strategies.

**Interventions:**

The exclusive intervention scenarios were 12 screening strategies based on different target population groups (age group 40–75 years, 50–75 years, 40–75 years age group at high risk of glaucoma, 50–75 years age group at high risk of glaucoma), screening methods (face-to-face screening and artificial intelligence-supported face-to-face screening) and screening frequencies for 40–75 years aged population (annual vs once every 5 years screening), in comparison to usual care scenario. The usual care scenario (current practice) implied opportunistic diagnosis by the ophthalmologists at higher levels of care.

**Primary and secondary outcomes:**

The primary outcome was the incremental cost–utility ratio for each of the screening strategies in comparison to usual care. The secondary outcomes were per person lifetime costs, lifetime out-of-pocket expenditures, life years and quality-adjusted life-years (QALYs) in all screening scenarios and usual care.

**Findings:**

Depending on the type of screening strategy, the gain in QALY per person ranged from 0.006 to 0.046 relative to usual care. However, the screening strategies, whether adjusted for specific age groups, patient risk profiles, screening methods or frequency, were not found to be cost-effective. Nonetheless, annual face-to-face screening strategies for individuals aged 40–75 years could become cost-effective in a scenario of strengthened public financing and provisioning, such that at least 67% of those seeking care for confirmatory diagnosis and treatment use government-funded facilities, in conjunction with 60% availability of medications at government hospitals.

**Conclusions:**

Enhancing continuity of care following screening through either strengthening of public provisioning or strategic purchasing of care could make glaucoma screening interventions not only cost-effective, but also potentially cost-saving.

**STRENGTHS AND LIMITATIONS OF THIS STUDY**

- The glaucoma screening strategies considered for this ‘what if’ economic analyses were conceptualised through targeted literature review, followed by consultation with policy-makers and ophthalmologists.
- The costs and quality of life of patients with glaucoma were acquired from Indian studies, making the analysis relevant for local decision-making.
- Since we accounted for current care-seeking behaviours, we provide a real-world estimate of the total cost of care, including out-of-pocket expenses and health system costs.
- The parameters related to treatment efficacy were obtained from studies conducted in other regions of the world, which may not be representative of Indian settings.
- Our economic analysis did not consider an integrated model of eye disorder screening, which is anticipated to be implemented in a real-life context.

## Introduction

 Glaucoma is a critical public health issue, being the second-leading cause of blindness, with vision loss from the condition being irreversible. Over 95% of individuals blinded by glaucoma reside in developing countries, primarily in Asia and sub-Saharan Africa.^1^ This high incident number of blindness cases is due to a combination of factors, including the substantial demographic weight of these regions, the high prevalence of glaucoma and limited access to healthcare. Furthermore, as demographic transition deepens and risk factors such as diabetes and hypertension become more prevalent, the burden of glaucoma is anticipated to rise, intensifying social and economic challenges at the population level.^2^

The WHO classifies glaucoma as one of the leading conditions responsible for 80% of avoidable blindness.^3^ Despite this, the disease receives limited attention and funding in developing countries, where only a small fraction of cases is diagnosed or treated. For instance, in India, glaucoma affects approximately 11.2 million individuals, accounting for 13% of blindness cases, yet the detection rate of the disease is as low as 10%.^4^ The limited focus on glaucoma can be attributed to several factors: the irreversible nature of vision loss, its asymptomatic early stages, less appealing therapeutic options available in its later stages and the lack of proven economic viability of systematic population-based early detection programmes in high-income countries.^5^,^7^

Nevertheless, existing economic literature from Asian countries supports the implementation of glaucoma screening programmes, highlighting their potential to facilitate early detection and management, thereby reducing the burden of visual impairment and blindness.^8 9^ For example, Tang *et al* found that combined screening for primary open-angle glaucoma (POAG) and primary angle-closure glaucoma (PACG) is likely to be cost-effective in the Chinese population.^8^ However, the results of this study may not be directly applicable to India due to differences in epidemiological parameters, healthcare systems and methodological approaches of analysis, such as study perspectives, discount rates and willingness-to-pay (WTP) threshold. Similarly, a study by John and Parikh indicated that the introduction of community screening for glaucoma could be cost-effective in India, based on a decision tree analysis. Nonetheless, this study considered only one-time treatment costs, and given the chronic nature of the disease, suggested that a lifetime horizon study would provide a more comprehensive evaluation.^9^

Given the current evidence gap, we aim to conduct an economic assessment of systematic population-based screening strategies for glaucoma, comparing them to the opportunistic diagnosis typically performed by ophthalmologists in India. This analysis is particularly pertinent in the context of India, considering the expansion of eye care packages and screening programmes at the primary healthcare (PHC) level under the Ayushman Bharat programme.^10^ Thus, this ‘what if’ analysis seeks to provide context-specific insights into the economic viability of various glaucoma screening strategies, thereby supporting decision-making on implementation models at primary care level.

Our study specifically examines the cost–utility of eight annual screening strategies based on different target population groups (age group 40–75 years, 50–75 years, 40–75 years age group at high risk of glaucoma, 50–75 years age group at high risk of glaucoma), and screening methods (face-to-face screening (F2F) and artificial intelligence-supported F2F screening (AI-based F2F)), relative to usual care scenario. We also evaluated screening in the 40–75 years age group (universal vs risk-based screening) at a frequency of once in 5 years. Additionally, we also assess the potential impact of implementation of population-based screening models on financial risk protection and overall costs of care for glaucoma.

## Methods

A systematic approach was employed to evaluate the cost–utility of screening strategies for glaucoma. The choice of cost–utility analysis was guided by the Indian reference case for conducting economic evaluations.^11^ In the absence of structured screening programmes for glaucoma in India, the first step involved defining strategies for introducing such a programme through consultations with eight stakeholders including three ophthalmologists, three policy-makers and two implementers. The feasibility of implementation at the primary care level was carefully considered during these consultations.

### Details of screening strategies and target population groups

Based on the consultations, it was assumed that screening services would be integrated with the existing services provided by PHCs in India. It was hypothesised that paramedical staff would conduct the screening for glaucoma and medical officers would be responsible for providing consultations at the PHCs. It was also assumed that accredited social health activists and community health officers would consistently engage in social mobilisation efforts to encourage participation in the screening programme.

In an F2F screening scenario, tonometry and retinal imaging would be performed for all the target population, while the oblique flashlight test would be only subjected to the population with high intraocular pressure. While AI-supported F2F screening (AI-based F2F) would be similar to F2F screening in terms of tonometry and flashlight test, the decision-making based on referrals based on retinal imaging would be guided by the deep learning algorithms embedded in the AI-enabled non-mydriatic fundus cameras. Population groups for screening were determined based on eye training manuals developed for PHC teams in India and expert consultations.^12^

We considered four population subgroups: population aged 40–75 years, population aged 50–75 years, population aged 40–75 years at high risk of glaucoma, population aged 50–75 years at high risk of glaucoma. Specifically, high-risk groups for glaucoma included individuals with diabetes and/or hypertension, as well as those with a family history of glaucoma. The population subgroups were representative of age-specific sociodemographic distribution in India. Further, we considered that population-based screening for the 40–75 years age group could be implemented at an annual or once in 5 years frequency, as guided by the expert consultation. Based on the combinations of different target population, mode and frequency of screening, 12 screening scenarios were contemplated for the economic evaluation.

Subsequently, given the chronic nature of the disease, a model-based analysis was conducted to evaluate these strategies in terms of lifetime costs and consequences compared with the current scenario in India. The current scenario implied opportunistic diagnosis by the ophthalmologists at higher levels of care. Glaucoma encompasses various subtypes, but for the purpose of this analysis, the evaluation focused on POAG and PACG, which together constitute 70% of glaucoma cases in India.^13^

#### Patient and public involvement

Patients and/or the public were not involved in the design, conduct, reporting or dissemination of this research.

### Model overview

An Excel-based Markov model integrated with a decision tree was developed to compare various glaucoma screening models. The model simulated a hypothetical cohort of 1000 individuals starting at age 40 or 50 years (depending on screening strategy), with a cycle length of 1 year. Lifetime health outcomes were assessed in terms of per person gain in life years (LYs), quality-adjusted life-years (QALYs). The evaluation undertook abridged societal perspective and incorporated both direct medical and non-medical costs, with future costs and outcomes discounted at an annual rate of 3%, in accordance with Indian methodological guidelines.^11^ We did not consider indirect costs in the analysis, as per the Indian guidelines.^11^ The incremental cost–utility ratio (ICUR) was calculated for each screening strategy relative to the usual care scenario. According to Indian economic evaluation standards, a screening strategy was considered cost-effective if its ICUR, expressed as incremental costs per QALY gained, was below the one-time GDP per capita for the year 2021–2022 (₹171 498) (₹=Indian National Rupee).^14^

### Model structure

**Figure 1:**
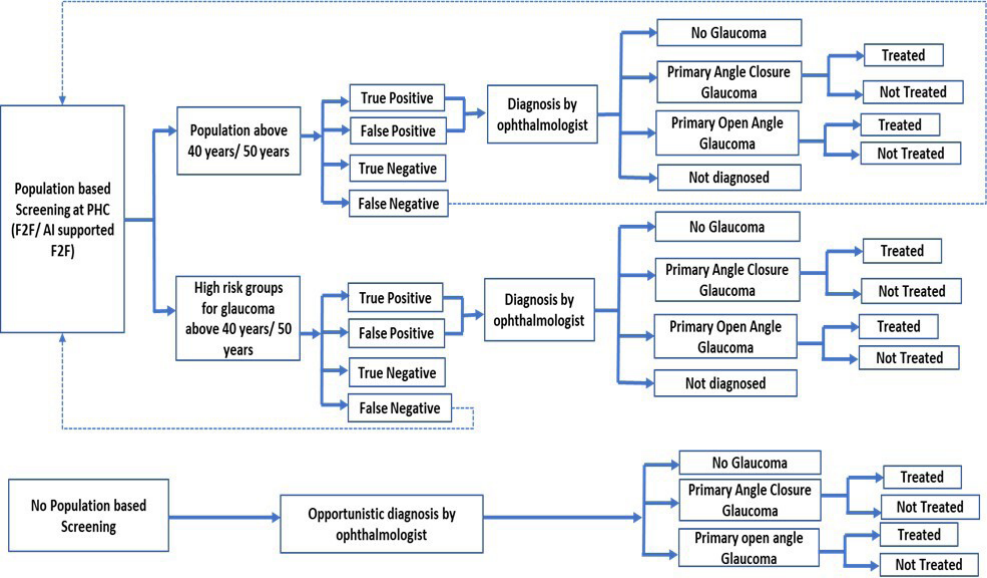
Decision tree. AI, artificial intelligence; F2F, face to face; PHC, primary healthcare.

A decision tree was developed to evaluate the screening of a cohort of 1000 individuals using one of the systematic strategies or usual care (figure 1). It was assumed that individuals suspected of having glaucoma would be referred to higher health facilities for confirmatory diagnosis by ophthalmologists, in accordance with standard treatment guidelines.^15^ Based on the diagnostic accuracy of the strategies and care-seeking behaviour, patients with glaucoma could be either ‘diagnosed’ or ‘not diagnosed’ and ‘treated’ or ‘not treated’ in each cycle.

**Figure 2:**
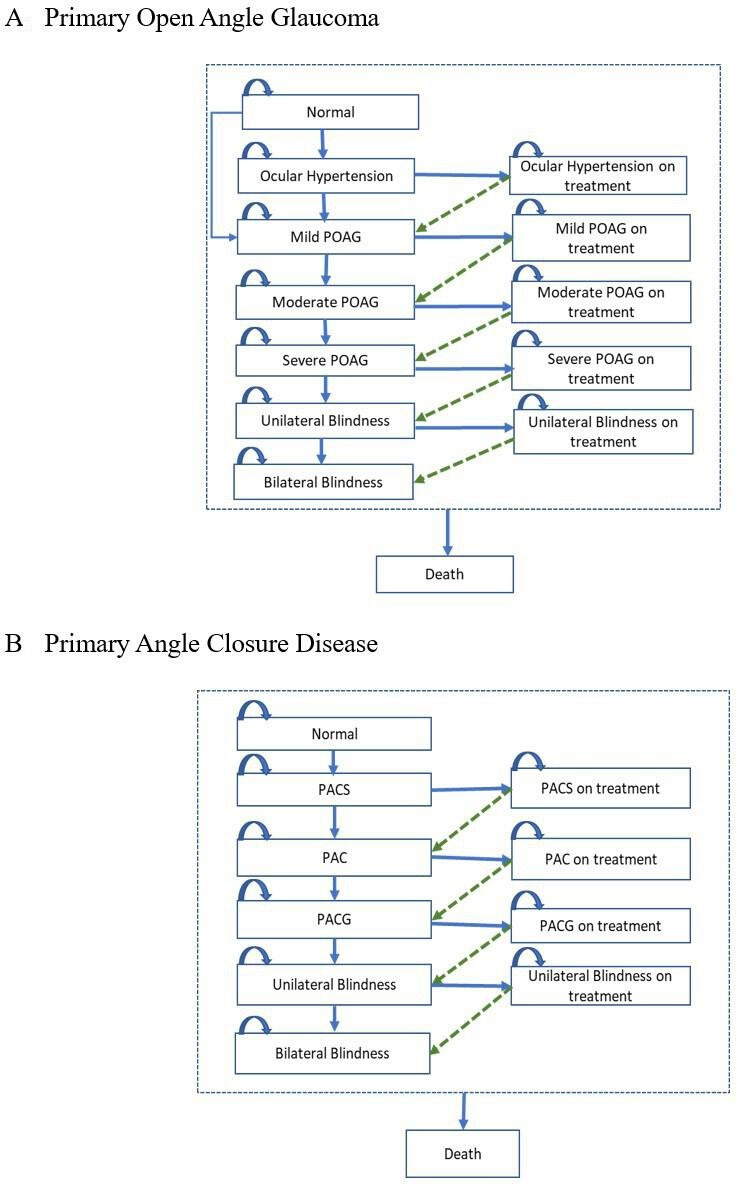
Schematic Markov model. PAC, primary angle closure; PACG, primary angle-closure glaucoma; PACS, primary angle closure suspect; POAG, primary open-angle glaucoma.

Subsequently, the natural progression of glaucoma was simulated using the Markov model (figure 2). For POAG, the model used the International Society of Geographical and Epidemiologic Ophthalmology (ISGEO) classification, which includes five stages: mild POAG, moderate POAG, severe POAG, POAG-related unilateral blindness and POAG-related bilateral blindness.^16^ Similarly, the ISGEO classification for primary angle-closure disease (PACD) was employed, encompassing five stages in addition to normal vision: primary angle closure suspect (PACS), primary angle closure (PAC), PACG without blindness, PACG-related unilateral blindness and PACG-related bilateral blindness.^16^ For both types of glaucoma, the model accounted for a reduced risk of progression to more severe states following treatment. The extent of this risk reduction was informed by published treatment efficacy data (online supplemental table S1).

### Model parameters

The coverage of screening was assumed to be 65% (online supplemental table S1). However, a 42% rate of loss to follow-up for confirmatory diagnosis at these higher centres was anticipated.^17 18^ Additionally, a 27.5% loss to follow-up for the initiation of treatment was factored into all scenarios involving confirmed diagnoses.^19^ According to Indian literature, in the usual care scenario (without population-based screening), 10% of individuals with glaucoma would consult an ophthalmologist, with 80% of these patients subsequently seeking treatment for glaucoma.^4 20^

The annual incidence rates for ocular hypertension (OHT) and POAG were derived from the Chennai Eye Disease Study, while the incidence of PACS was obtained from the Andhra Pradesh Eye Disease Study.^21 22^ The probability of disease progression was extracted from published literature (online supplemental table S1). Age-specific mortality rates were calculated using the Sample Registration System life tables.^23^ A mortality multiplier of 2.34 for blindness was applied, based on HRs reported in published research.^24^

The sensitivity and specificity of the screening tests were determined from published studies (online supplemental table S1). Diagnostic accuracy for OHT and POAG was based on tonometry and non-mydriatic fundus camera images, respectively. For the screening of PAC or PACG, the combined sensitivity and specificity of the oblique flashlight test and tonometry were used, assuming they were performed in series. It was also assumed that 22% of retinal images would be ungradable, based on data from a national study.^25^ The diagnoses made at higher centres using gold standard tests were considered to accurately reflect the true health state.

### Cost of screening and treatment

The evaluation of screening and treatment costs encompassed both health system costs and out-of-pocket expenditure (OOPE) borne by patients. To estimate the costs for various screening scenarios, the data from studies included in the National Health System Cost Database of India were used.^26^ The cost calculations were adjusted based on different screening rates, varying variable costs, the mean population served by each PHC, the additional inputs required for service delivery and adjusted for inflation to convert all costs to 2022 (online supplemental table S2, online supplemental figure S1). For AI-supported screening, the average cost per scan was determined through three rounds of consultations with major technology suppliers in India, reflecting a health systems perspective. Additionally, costs associated with training human resources and conducting quarterly supervision were factored into the overall cost estimation (online supplemental section S2).

The treatment costs for glaucoma were based on Indian clinical guidelines.^15^ The treatment costs included the cost of consultations, diagnostic tests, medicines/surgical costs and non-medical costs. For POAG, the treatment options included antiglaucoma medications and trabeculectomy. For PACD, the treatments comprised iridotomy, antiglaucoma medications and trabeculectomy. It was assumed that patients undergoing trabeculectomy would still require antiglaucoma medications. According to the guidelines, patients would need a series of follow-up visits annually, depending on their health states.^15^

Information on health system costs for treatment at public facilities was obtained from the National Health Systems Cost Database.^26^ Costs associated with diagnostics, medical and surgical management of glaucoma at private health facilities were gathered from available open sources (online supplemental table S1). When multiple data sources were available for private sector costs, the mean estimate was used in the economic model to ensure generalisability. Data on care-seeking patterns and non-medical costs were sourced from the National Sample Survey, which collects data on morbidity and OOPE from 555 114 households spread over every district of the country.^27^

### Health state utility values

A facility-based cross-sectional study was undertaken to evaluate the quality of life in glaucoma patients. This study involved the recruitment of 297 patients who attended the ophthalmic outpatient department at a tertiary care hospital in northern India. Data collection was carried out through F2F interviews using the EuroQoL Five-Dimensions Five-Levels (EQ-5D-5L) tool. The mean utility scores, along with their standard errors, were computed using the EQ-5D-5L Indian value set.^28^ Furthermore, the combined utility values for various stages of glaucoma, alongside the presence of hypertension, diabetes or both conditions, were determined using a multiplicative method.^29^ The utility values associated with hypertension and/or diabetes were obtained from a previously published Indian study (online supplemental section S3).^30^

### Sensitivity analysis

Following the assessment of lifetime costs and health benefits, we computed the ICURs for each screening strategy relative to the current standard practice in India. To address uncertainties in parameter values, we conducted both deterministic and probabilistic sensitivity analyses (PSA). This was done to account for potential variations in prevalence, disease progression, diagnostic accuracy, compliance and utility values. Where ranges were not available in the published literature, parameters were varied by 10%, and due to significant regional cost differences, a broader variation of 50% was applied.

Each parameter was assigned an appropriate distribution based on its characteristics: gamma distribution was applied to cost parameters, while beta distribution was used for quality-of-life estimates and other parameters reported as proportions or probabilities (online supplemental table S1). We performed 1000 iterations of Monte Carlo simulations to analyse the ICURs.

We conducted a scenario analysis to model a case of public sector strengthening for glaucoma care through public provisioning or publicly financed and strategically purchased care. We analysed the ICUR values at different proportions of glaucoma suspected population (who are likely to visit higher facilities considering the current care-seeking behaviour in India) accessing public facilities or government-funded private institutions for confirmatory diagnosis and treatment, as a result of shift from the private sector. We did not consider different rates of loss-to-follow-up for diagnosis since it would require support interventions, such as counselling, which were not considered and hence not costed in the model. We also analysed the effect of the availability of free medicines at the government facilities on the ICUR values. A two-way sensitivity analysis was run by varying the proportions of population accessing public facilities and differential availability of free medicines at the public facilities.

We also assessed the impact of the screening programme on lifetime OOPE and the total direct cost of care to the health system and the population from the model. Rates of shift of population from private to public facilities for diagnosis and treatment, and availability of free medicines were varied to gauge their impact on OOPE and the total cost of care.

## Results

### Incremental costs, outcomes and cost–utility ratios

Table 1 details the mean lifetime costs and health outcomes in terms of discounted LYs and QALYs per patient in different glaucoma screening scenarios. In comparison to usual care, annual AI-supported F2F screening in the 40–75 years old cohort resulted in the highest gain of 0.0062 (0.0059 to 0.0066) LYs and 0.0482 (0.0466 to 0.0496) QALY per person; however, with an incremental cost of ₹17 050 (16 786 to 17 314). The F2F annual screening strategy in the 40–75 years age group yielded the lowest ICUR of ₹338099 per QALY gained and led to a 32% reduction in lifetime incidence of blindness. The ICUR values across all strategies surpassed the WTP threshold, indicating that the interventions are not cost-effective when compared with usual care (table 1).

**Table 1:** Discounted mean life years, QALY and cost per person and incremental cost–utility ratio

Strategies	Life years per person	QALY per person	Lifetime cost per person (₹)	Incremental cost (₹) per QALY gained
Annual screening (40–75 years)				
Usual care	19.6901 (19.6866–19.6935)	17.3186(17.2969–17.3409)	6,908(6,629–7,187)	
High-risk population (F2F)	19.6929(19.6894–19.6962)	17.3454(17.3235–17.3674)	17,174(16,964–17,384)	412,528(193,521–1,304,829)
High-risk population (AI)	19.6929(19.6895–19.6964)	17.3461(17.5503–17.9285)	18,171(12,532–25,822)	455,855(206,918–1,511,437)
40–75 years population (F2F)	19.6963(19.6929–19.6997)	17.3663(17.3444–17.3972)	22,103(21,881–22,325)	338,099(164,258–1,267,897)
40–75 years population (AI)	19.6963(19.6929–19.6998)	17.3670(17.3451–17.3889)	23,959(23,728–24,190)	374,287(177,222–1,338,827)
Annual screening (50–75 years)				
Usual care	15.6041(15.6001–15.6081)	14.1445(14.1717–14.1771)	5,061(4,851–5,271)	
High-risk population (F2F)	15.6047(15.6007–15.6087)	14.1727 (14.1592–14.1863)	11,007(10,841–11,173)	413,619(155,709–1,899,707)
High-risk population (AI)	15.6047(15.6008–15.6087)	14.1732(14.1596–14.1868)	11,964(11,798–12,130)	473,598(177,243–2,192,715)
40–75 years population (F2F)	15.6068(15.6027–15.6107)	14.1906(14.1771–14.2042)	17,176(17,004–17,347)	385,918(158,760–1,343,505)
40–75 years population (AI)	15.6067(15.6027–15.6109)	14.1911(14.1775–14.2047)	18,716(18,536–18,896)	434,713(174,193–1,575,070)
Once in 5 years screening (40–75 years)				
Usual care	19.6901 (19.6867–19.6936)	17.3189(17.2969–17.3409)	6,908(6,629–7,187)	
High-risk population (F2F)	19.6910(19.6875–19.6944)	17.3037(17.3477–17.3628)	9,621(9,381–9,860)	432,630(185,358–1,446,353)
High-risk population (AI)	19.6911(19.6876–19.6945)	17.3261(17.3041–17.3480)	10,150(9,917–10,383)	507,474(197,713–1,969,644)
40–75 years population (F2F)	19.6922(19.6887–19.6956)	17.3328(17.3109–17.3548)	11,225(11,002–11,448)	344,697(154,778–1,091,446)
40–75 years population (AI)	19.6923(19.6888–19.6957)	17.3332(17.3112–17.3551)	11,897(11,679–12,115)	393,870(160,961–1,184,671)

Values in parenthesis present values at 95% confidence intervalsCIs derived using Monte Carlo simulation method. F2F: Face-to-face screening, AI: Artificial intelligence supported face-to-face screening. ₹: Indian National Rupee

AIartificial intelligenceF2Fface to faceQALYquality-adjusted life-year

The PSA highlighted that the likelihood of the screening strategies being cost-effective at the current WTP threshold ranged from 0.6% to 4% (table 2). As the WTP threshold increases, the probability of these strategies being deemed cost-effective compared with usual care also increases.

**Table 2:** Probability of screening strategies being cost-effective compared with usual care at different willingness-to-pay thresholds*

Strategies	% probability of being cost-effective	
	WTP=₹171 498(One-time GDP per capita)	WTP=₹514 494(Three-time GDP per capita)
Annual screening (40–75 years)		
High-risk population (F2F)	1.1%	70.4%
High-risk population (AI)	0.6%	62.6%
40–75 years population (F2F)	2.6%	82.3%
40–75 years population (AI)	1.2%	75.6%
Annual screening (50–75 years)		
High-risk population (F2F)	2.6%	63.6%
High-risk population (AI)	1.4%	56.0%
40–75 years population (F2F)	2.8%	71.0%
40–75 years population (AI)	1.3%	63.3%
Once in 5 years screening (40–75 years)		
High-risk population (F2F)	1.2%	64.5%
High-risk population (AI)	0.8%	50.5%
40–75 years population (F2F)	4.0%	82.0%
40–75 years population (AI)	2.9%	73.0%

* Probability of cost-effectiveness computed through probabilistic sensitivity analysis using Monte Carlo simulation method.

AIartificial intelligenceF2Fface to faceWTPwillingness to pay

The patterns of healthcare utilisation and the availability of medications in government facilities had a noteworthy impact on the ICUR values. Analysis indicated that a shift in the use of government-funded facilities, rising from 37.8% to 55% for outpatient care and from 32% to 55% for inpatient care, among screened individuals who seek confirmatory diagnosis and treatment at higher facilities, would render the once-every-5 years F2F screening strategy for the 40–75 years age group cost-effective, with an ICUR of ₹160 180. Moreover, if the availability of glaucoma medications at government-funded institutions were to increase from 20% to 60%, coupled with the aforementioned shift to government-funded facilities, all once-every-5 years screening strategies would become cost-effective.

**Figure 3:**
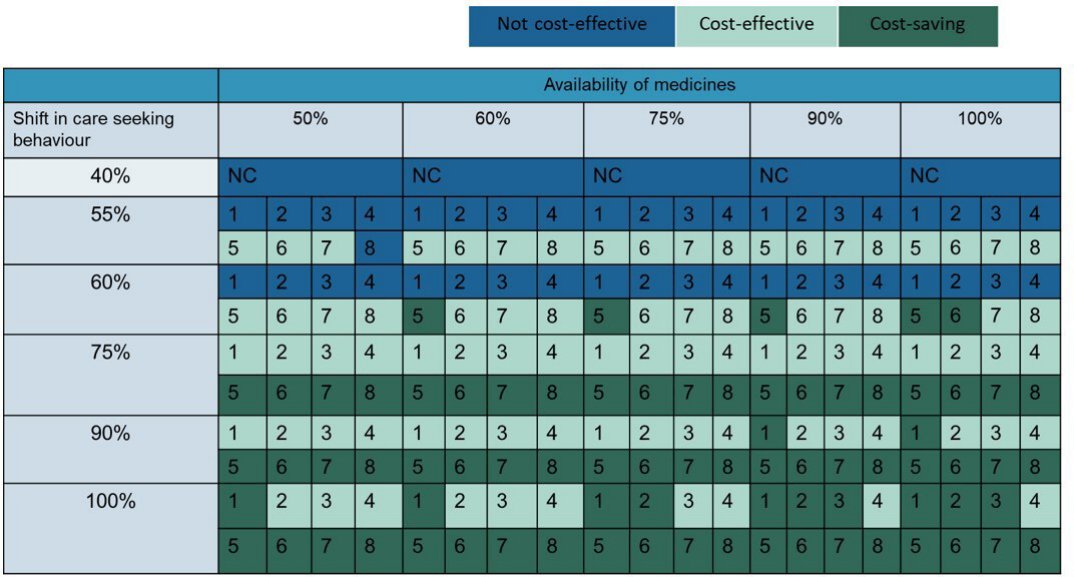
Effect of care-seeking behaviour and availability of medicines on cost-effectiveness of glaucoma screening strategies. 1–4: Annual screening strategies in 40–75 years age group 1: F2F screening in high-risk population; 2: AI-supported F2F screening in high-risk population; 3: F2F screening in all population; 4: AI supported F2F screening in all population 5–8: Once in 5 years screening strategies in 40–75 years age group 5: F2F screening in high-risk population; 6: AI-supported F2F screening in high-risk population; 7: F2F screening in all population; 8: AI supported F2F screening in all population. AI, artificial intelligence; F2F, face to face; NC, not cost-effective.

Annual F2F screening strategies for individuals aged 40–75 years transition to be cost-effective if the utilisation of government-funded facilities reaches at least 67%, in conjunction with a 60% availability of medications. Conversely, annual AI-based F2F screening strategies for the same age group become cost-effective if 75% of the screened glaucoma-suspected population, who are likely to seek care, use government-funded facilities for confirmatory diagnosis and treatment, alongside a minimum free medication availability of 50%. The results from the two-way sensitivity analysis are presented in figure 3.

### Effect of strengthening continuity of care on OOPEs and overall cost of care

**Figure 4:**
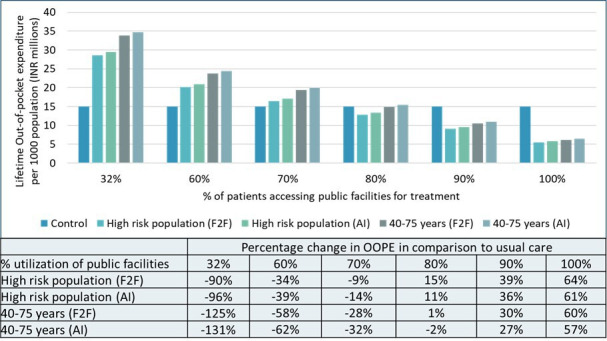
Effect of change in care-seeking pattern on out-of-pocket expenditure. Negative sign indicates higher OOPE in comparison to usual care. AI, artificial intelligence; F2F, face to face; OOPE, out-of-pocket expenditure.

We found that if 90% of the screened suspected glaucoma cases who seek confirmatory diagnosis and treatment at higher facilities use government-funded facilities, there would be a reduction of 27%–39% in OOPE at per-capita level (figure 4).

We observed that merely a shift in care-seeking pattern at population level did not lead to a reduction in overall cost of care, except if the F2F screening is limited to population at high risk of glaucoma (online supplemental table S3). However, achieving a minimum of 50% availability of medications at health facilities, combined with the utilisation of government-funded facilities by 90% of suspected glaucoma cases seeking care (after consideration of loss-to-follow-up of 42% among the referred cases), would result in a reduction in overall care costs for alternative annual screening strategies. Specifically, this reduction applied to both AI-based screening for high-risk glaucoma populations and F2F screening for the 40–75 age group and would yield improved health outcomes compared with the standard care scenario.

## Discussion

India is currently experiencing demographic shifts characterised by an ageing population and a transition from infectious diseases to non-communicable chronic diseases.^31^ This transition has led to an increased demand for healthcare services for chronic conditions amidst constrained health resources. In this context, there is an urgent need to develop an efficient disease management model that emphasises PHC services, including screening for chronic diseases, rather than relying solely on hospital care. Unlike clinical interventions such as pharmaceuticals and medical devices, which directly impact disease pathogenesis, PHC offers exponential benefits by addressing risk factors to prevent the onset and progression of diseases, enhancing access to care, ensuring continuity of care, and providing financial risk protection.^32^

In our model-based economic evaluation, we assessed systematic screening strategies for glaucoma at the PHC level against the usual care scenario, which involves opportunistic detection by ophthalmologists, from an abridged societal perspective. Our analysis indicates that none of the annual screening strategies, whether tailored to specific age groups, patient risk profiles or screening methods, proved cost-effective compared with the usual care scenario, given the WTP threshold of one-time GDP per capita (₹171 498) in India. Furthermore, we found that increasing the screening interval to once every 5 years led to a reduction of approximately 50% in lifetime costs per person relative to annual screening strategies. Nonetheless, despite this cost reduction, the screening strategies remained cost-ineffective compared with the usual care approach.

Our study’s results are consistent with those observed in numerous high-income countries, which indicate that the screening is likely to be cost-ineffective in low-income and-middle-income countries, despite the higher prevalence of glaucoma and lower healthcare costs. In the UK, research has indicated that the ICERs for population-based glaucoma screening exceed the threshold of £30 000 per QALY, rendering it less cost-effective compared with not implementing a screening programme (online supplemental table S4).^6 7^ This may be attributed to low prevalence of disease, high screening costs and high rate of opportunistic diagnosis in the counterfactual scenarios, leading to lower yield in health outcomes. In the USA, opportunistic diagnosis of glaucoma during routine ophthalmologic examinations is considered cost-effective relative to scenarios involving no diagnosis or treatment.^33^ However, this study did not evaluate population-based screening. A recent review by the US Preventive Services Task Force underscored a lack of comprehensive evidence to precisely evaluate the costs and benefits of glaucoma screening.^34^ Consequently, major health organisations, such as the US Preventive Services Task Force and the UK National Screening Committee, do not endorse population-wide glaucoma screening.^35 36^

In contrast, studies conducted in China, Canada and Spain have concluded that organised screening programmes are a cost-effective alternative to opportunistic disease detection.^8 37 38^ Although there are notable differences between these studies and our analysis, such as variations in screening strategies, treatment pathways and methodological approaches to analysis (online supplemental table S3), it is noteworthy that the cost of treatment in all these studies was based on costs incurred in government facilities. This approach may be appropriate in countries with robust public healthcare systems or systems where costs are controlled by the government. However, in a country like India, which has a mixed healthcare system with high extent of OOPE, such analyses may not accurately reflect the true cost of care, potentially leading to an underestimation of actual healthcare costs.

Our findings suggest that enhancing continuity of care through either strengthening of public provisioning or strategic purchasing of care, combined with change in treatment-seeking patterns at population level, could render glaucoma screening interventions not only cost-effective, but also potentially cost-saving. Specifically, if 55% of the screened suspected individuals who are likely to seek care at higher facilities, considering current treatment-seeking behaviour, that is, 32% of referred cases, visit government-funded institutions for care, at least one of the glaucoma screening strategies becomes cost-effective. Furthermore, utilisation of government-funded health facilities by a minimum of 46% of the referred cases starts to reduce OOPE at population level. This underscores the importance of improving continuity of care through integrated care systems as a strategy to achieve financial risk protection and enhance the efficiency of healthcare interventions.

Our analysis indicated that the additional healthcare costs associated with a glaucoma screening programme could not be offset by reductions in disease progression. This finding aligns with research from China, which reported that the incremental costs of screening, compared with no screening, amounted to US$434 903 over a 15-year period, translating to US$1464 per PACG case prevented at any stage.^39^

However, we found that if 50% of referred cases visit government-funded institutions after screening and 50% of these glaucoma patients requiring AGM receive these medications free of charge, there can be a reduction in overall cost of care associated with screening, diagnosis and treatment of the disease at societal level. In the current scenario, the essential medicine lists (EMLs) in India currently include only pilocarpine and timolol under the category of antiglaucoma medications at secondary and tertiary institutions, with no antiglaucoma medicines at PHC facilities.^40 41^ This has translated into a low number of glaucoma patients receiving free medicines at the government institutions.^27^ The government has, however, taken initiatives to improve the availability of subsidised, generic medicines through Jan Aushadhi Kendras under the Pradhan Mantri Bhartiya Jan Aushadhi Pariyojana.^42^ However, there is a need to improve awareness about the scheme as well as disseminate facts about quality and costs of generic medicines.^43^ Health systems strengthening efforts through expansion of EML or provision of medicines through subsidised outlets are likely to not only improve the efficiency of screening programmes for glaucoma but will also reduce the overall cost of care at societal level, in comparison to the current scenario.

Our findings diverge from those of a previous decision-tree-based study conducted in India, which only accounted for the one-time cost of glaucoma treatment and assumed a 0.24 increase in utility value associated with glaucoma treatment compared with no treatment.^9 44^ In contrast, our analysis incorporated lifetime costs and health outcomes related to the disease and did not factor in any change in utility value resulting from treatment, based on our primary data analysis. This accords with the biological plausibility of the disease, where glaucoma is known to cause irreversible loss of vision, which is the main determinant of utility in patients with glaucoma.^8 45 46^

### Model validation

We estimated the annual incidence rates of OHT, POAG and PACSs at 370, 489 and 530 per 100 000 persons. Furthermore, the ratio between blindness due to PACD and POAG was 1:1 for unilateral blindness. PACD caused 2.2 times the proportion of bilateral blindness than POAG. These findings were in lines with Indian epidemiological studies.^47^

The cost of care per person in private facilities was 2.8 times the cost of care in public facilities. This was in line with the research findings by Garg *et al*, which calculated the ratio between cost of care by private hospitals and district hospital for outpatient care at 2.5.^48^ Additionally, OOPE per person in private facilities was 6.5 times the OOPE per person in public facilities, in the usual care scenario. This aligned with the findings from the recent National Sample Survey round, which estimated the ratio of OOPE in private facilities to public facilities for outpatient care at 6.72.^27^

### Strengths and limitations

Our study has several notable strengths. First, we evaluated several possible screening strategies, which could be considered in Indian PHC system in consultation with policy-makers and ophthalmologists. Second, we conducted primary data collection to assess the quality of life associated with different health states and used the EQ-5D-5L Indian value set to calculate utility index values. Third, we accounted for coverage rates, drop-out rates and treatment compliance based on existing literature, as well as the rate of non-gradability of fundus images and varying costs of screening corresponding to different screening frequencies. Fourth, we considered current care-seeking behaviours to estimate the total cost of care, including OOPEs and health system costs. Given the lack of standardisation in the cost of AI technology for glaucoma, we consulted stakeholders to obtain market quotations from major technology suppliers in India and used an average estimate for modelling purposes. We followed the Consolidated Health Economic Evaluation Reporting Standards checklist to ensure that the study methods and findings are reported in a comprehensive and standardised manner (online supplemental table S5).

Our study results should be seen in light of a few limitations due to paucity of literature. First, as a modelling study, the predictive accuracy relies on parameters used in the Markov model. There are potential risks that treatment efficacy parameters, parameters pertaining to relative risk of glaucoma in different population subgroups, obtained from existing studies conducted in other regions may not be representative of Indian settings. However, the parameters did not alter the direction of results in one-way sensitivity analysis. Second, we limited our analysis to POAG and PACD, which constitute 70% of the glaucoma in India.^12^ Third, we did not vary the rates of incomplete follow-up and non-compliance with treatment, to avoid model complexity. Fourth, the study was performed considering the peculiarities of healthcare system in India and some of the results may not be applicable to other environments. Our analysis did not account for the additional benefits of screening, in terms of detection of multiple eye conditions. Future research should investigate the cost-effectiveness of routine and tailored ophthalmologic assessments designed to detect multiple eye conditions.

Our study offers crucial insights for decision-makers on optimising glaucoma screening models. Although the Indian government is exploring the expansion of eye care services within a comprehensive PHC framework, implementing a glaucoma screening programme may not be cost-effective in the current context of care seeking behaviours skewed towards private sector. The study highlights the need to prioritise enhancing continuity of care by fortifying connections between PHC and hospitals. Strengthening PHC oriented health systems through adequate financing for diagnosis and treatment within the government funded institutions is expected to not only improve the efficiency of glaucoma screening programmes but also reduce the overall cost of glaucoma care at societal level.

## supplementary material

10.1136/bmjopen-2024-098113 online supplemental file 1

## Data Availability

All data relevant to the study are included in the article or uploaded as supplementary information.
