# Supplementary material for: Seizing the silent vision loss: cost–utility analysis of population-based glaucoma screening in India
Source: BMJ Open. 2025 Apr 3;15(4):e098113. doi: 10.1136/bmjopen-2024-098113 (PMC11969579; doi:10.1136/bmjopen-2024-098113)
Supplement: online supplemental file 1 [file bmjopen-15-4-s001.docx]

**Seizing the Silent Vision Loss: Cost-Utility Analysis of Population-Based Glaucoma Screening in India**

**Supplement**

**Contents**

[**Section S1. Input parameters** 2](#_Toc192705048)

[**Section S2. Details of costing of screening services** 7](#_Toc192705049)

[**Section S3. Calculation of utility values in co-morbidities** 10](#_Toc192705050)

[**Section S4. Details of literature on economic evaluation of glaucoma screening** 11](#_Toc192705051)

[**Section S5. Compliance to CHEERS Checklist** 13](#_Toc192705052)

[Table S1. Input parameters 2](#_Toc192705056)

[Table S2. Per person cost of screening according to variations in screening coverages (₹) 9](#_Toc192705057)

[Table S3. Change in overall cost of care with varying availability of medicines at government-funded health facilities (₹ millions) 10](#_Toc192705058)

[Table S4. Evidence from existing literature on economic evaluation of glaucoma screening (1995-May 2024) 11](#_Toc192705059)

[Table S5. CHEERS Checklist 13](#_Toc192705060)

# **Section S1. Input parameters**

Table S1. Input parameters

| Epidemiological Parameters |  |  |  |  |
| --- | --- | --- | --- | --- |
|  | Base Case Value | Standard Error | Distribution | Reference |
| Prevalence at 40 years |  |  |  |  |
| OHT | 0.0032 | 0.0032 | Beta | [1] |
| POAG | 0.0021 | 0.0026 |  |  |
| PACD | 0.0021 | 0.0010 |  | [2] |
| Prevalence at 50 years |  |  |  |  |
| OHT | 0.0043 | 0.0002 | Beta | [1] |
| POAG | 0.0069 | 0.0004 |  |  |
| PACD | 0.0046 | 0.0003 |  | [2] |
| Proportion of disease in tertiary hospitals |  |  |  |  |
| Mild POAG | 0.27 | 0.0156 | Beta | [3], [4] |
| Moderate POAG | 0.34 | 0.0196 |  |  |
| Severe POAG | 0.39 | 0.0225 |  |  |
| PACS | 0.18 | 0.0104 |  | [5] |
| PAC | 0.12 | 0.0069 |  |  |
| PACG | 0.7 | 0.0404 |  |  |
| Proportion of disease in community |  |  |  |  |
| Mild POAG | 0.73 | 0.0422 | Beta | [6] |
| Moderate POAG | 0.23 | 0.0134 |  |  |
| Severe POAG | 0.04 | 0.0021 |  |  |
| Odds of OHT |  |  |  |  |
| In diabetic population | 1.7 | 0.9815 | Uniform | [7] |
| People with family history | 1.5 | 0.0866 |  |  |
| In hypertensive population | 1.32 | 0.0762 |  | [8] |
| Relative risk of OAG onset |  |  |  |  |
| In diabetic population | 1.38 | 0.0797 | Uniform | [9] |
| People with family history | 4.25 | 0.1328 |  |  |
| In hypertensive population | 1.1 | 0.0635 |  | [10] |
| Odds of PACD |  |  |  |  |
| In diabetic population | 1.2 | 0.0692 | Uniform | [10], [11], [12] |
| In hypertensive population | 1 | 0.0577 |  |  |
| People with family history | 1.2 | 0.0699 |  | [12] |
| Incidence of POAG |  |  |  |  |
| 40-49 years | 0.0018 | 0.0006 | Beta | [13] |
| 50-59 years | 0.0042 | 0.0008 |  |  |
| 60-69 years | 0.0075 | 0.0012 |  |  |
| 70+ years | 0.0076 | 0.0015 |  |  |
| Incidence of PACS |  |  |  |  |
| 40-49 years | 0.0066 | 0.0004 | Beta | [14] |
| 50-59 years | 0.0055 | 0.0003 |  |  |
| 60+ years | 0.0039 | 0.0002 |  |  |
| Prevalence of hypertension and diabetes at 40 years |  |  |  |  |
| Hypertension | 0.224 | 0.0259 | Beta | [15] |
| Diabetes | 0.072 | 0.0083 |  | [16] |
| Prevalence of hypertension and diabetes at 50 years |  |  |  |  |
| Hypertension | 0.398 | 0.0460 | Beta | [15] |
| Diabetes | 0.128 | 0.0148 |  | [16] |
| Annual incidence-hypertension | 0.0826 | 0.0095 |  | [17] |
| Annual incidence-diabetes | 0.0217 | 0.0025 |  | [18] |
| Transition probabilities |  |  |  |  |
| Normal to OHT | 0.0036 | 0.0002 | Beta | [19] |
| OHT to mild POAG | 0.02 | 0.0012 |  | [20] |
| Mild to Moderate POAG | 0.149 | 0.0641 |  | [21],[22],[23] |
| Moderate to Severe POAG | 0.0796 | 0.0231 |  | [21], [22] |
| Severe POAG to unilateral blindness | 0.0263 | 0.0439 |  | [21] |
| Unilateral to bilateral blindness | 0.0719 | 0.0041 |  |  |
| Treated OHT to mild POAG | 0.008 | 0.0004 |  | [20] |
| Treated mild to moderate POAG | 0.073 | 0.0042 |  | [24] |
| Treated moderate to severe POAG | 0.0517 | 0.0029 |  | [22] |
| Treated severe POAG to unilateral blindness | 0.0149 | 0.0055 |  | [25],[26],[27] |
| Treated unilateral blindness to bilateral blindness | 0.0156 | 0.0009 |  | [27] |
| PACS to PAC | 0.0485 | 0.0028 |  | [28] |
| PAC to PACG | 0.0766 | 0.0044 |  |  |
| PACG to unilateral blindness | 0.2086 | 0.0120 |  | [21],[29] |
| Unilateral blindness to bilateral blindness | 0.1797 | 0.0103 |  |  |
| Treated PACS to PAC | 0.0216 | 0.0012 |  | [30],[31],[32] |
| Treated PAC to PACG | 0.0094 | 0.0005 |  | [30],[31] |
| Treated PACG to unilateral blindness | 0.007 | 0.0005 |  | [33] |
| Treated Unilateral blindness to bilateral blindness | 0.0387 | 0.0022 |  | [21] |
| Mortality parameters |  |  |  |  |
| 40-44 | 0.004 | 0.0002 | Beta | [34] |
| 45-49 | 0.005 | 0.0003 |  |  |
| 50-54 | 0.008 | 0.0005 |  |  |
| 55-59 | 0.013 | 0.0008 |  |  |
| 60-64 | 0.019 | 0.0011 |  |  |
| 65-69 | 0.029 | 0.0016 |  |  |
| 70-74 | 0.044 | 0.0025 |  |  |
| 75-79 | 0.065 | 0.0028 |  |  |
| Mortality multiplier- blindness | 2.34 | 0.270 |  | [35] |
| Diagnostic parameters (non-mydriatic fundus camera and tonometer) |  |  |  |  |
| **Non-mydriatic fundus camera** |  |  |  |  |
| Sensitivity | 0.62 | 0.0841 | Normal | [36],[37] |
| Specificity | 0.85 | 0.0289 |  |  |
| **Tonometer** |  |  |  |  |
| Sensitivity | 0.50 | 0.0731 | Normal | [38] |
| Specificity | 0.98 | 0.0036 |  |  |
| **Oblique flashlight and tonometer** |  |  |  |  |
| Sensitivity | 0.425 | 0.0491 | Normal | [39] |
| Specificity | 0.997 | 0.0006 |  |  |
| **Artificial intelligence enabled non-mydriatic fundus camera** |  |  |  |  |
| Sensitivity | 0.937 | 0.1086 | Normal | [40] |
| Specificity | 0.856 | 0.1486 |  |  |
| **Diagnosis by ophthalmologist** |  |  |  |  |
| Sensitivity | 1 |  |  | Expert Consultation |
| Specificity | 1 |  |  |  |
| **Healthcare utilization rates** |  |  |  |  |
| Screening coverage | 0.65 | 0.0683 | Normal | [41] |
| Diagnosis rate in usual care | 0.1 | 0.0882 |  | [2], [42] |
| Loss-to-follow-up for diagnosis in screening scenario | 0.42 | 0.0833 |  | [43],[44] |
| Treatment coverage in screening scenario | 0.725 | 0.1146 |  | [45] |
| Treatment coverage in usual care | 0.8 | 0.0458 |  | [2], [46] |
| Treatment- OHT (screening scenario) | 0.5 | 0.0577 |  | Expert consultation |
| Treatment adherence after initiation of treatment | 0.5 | 0.0577 |  | [47] |
| Discount Rate |  |  |  |  |
| Benefits | 0.03 | - |  | [48] |
| Costs | 0.03 | - |  |  |
| Utility values |  |  |  |  |
| **In hypertensive patients** |  |  |  |  |
| No glaucoma | 0.890 | 0.0514 | Beta | [49], [50] |
| OHT | 0.801 | 0.0462 |  |  |
| Mild POAG | 0.712 | 0.0411 |  |  |
| Moderate POAG | 0.668 | 0.0385 |  |  |
| Severe POAG | 0.632 | 0.0365 |  |  |
| PACS | 0.846 | 0.0488 |  |  |
| PAC | 0.801 | 0.0462 |  |  |
| PACG | 0.668 | 0.0385 |  |  |
| Unilateral Blindness | 0.418 | 0.0242 |  |  |
| Bilateral Blindness | 0.231 | 0.0134 |  |  |
| **In diabetic patients** |  |  |  |  |
| No glaucoma | 0.760 | 0.0439 | Beta | [49], [50] |
| OHT | 0.684 | 0.0395 |  |  |
| Mild POAG | 0.608 | 0.0351 |  |  |
| Moderate POAG | 0.570 | 0.0329 |  |  |
| Severe POAG | 0.540 | 0.0312 |  |  |
| PACS | 0.722 | 0.0417 |  |  |
| PAC | 0.684 | 0.0395 |  |  |
| PACG | 0.570 | 0.0329 |  |  |
| Unilateral Blindness | 0.357 | 0.0206 |  |  |
| Bilateral Blindness | 0.198 | 0.0114 |  |  |
| **In diabetic and hypertensive patients** |  |  |  |  |
| No glaucoma | 0.680 | 0.0393 | Beta | [49], [50] |
| OHT | 0.609 | 0.0351 |  |  |
| Mild POAG | 0.541 | 0.0312 |  |  |
| Moderate POAG | 0.507 | 0.0293 |  |  |
| Severe POAG | 0.480 | 0.0277 |  |  |
| PACS | 0.643 | 0.0371 |  |  |
| PAC | 0.609 | 0.0351 |  |  |
| PACG | 0.507 | 0.0293 |  |  |
| Unilateral Blindness | 0.318 | 0.0184 |  |  |
| Bilateral Blindness | 0.176 | 0.0102 |  |  |
| **In people without diabetes and hypertension** |  |  |  |  |
| No glaucoma | 1 | 0.0577 | Beta | [50] |
| OHT | 0.90 | 0.0520 |  |  |
| Mild POAG | 0.80 | 0.0462 |  |  |
| Moderate POAG | 0.75 | 0.0433 |  |  |
| Severe POAG | 0.71 | 0.0410 |  |  |
| PACS | 0.95 | 0.0548 |  |  |
| PAC | 0.90 | 0.0519 |  |  |
| PACG | 0.75 | 0.0433 |  |  |
| Unilateral Blindness | 0.47 | 0.0271 |  |  |
| Bilateral Blindness | 0.26 | 0.0150 |  |  |
| **Screening costs** |  |  |  |  |
| Screening (F2F) 40-75 years | 102 | 29.449 | Gamma | Authors analysis using [51] |
| Screening (F2F) high risk population | 132 | 38.105 |  |  |
| Screening (F2F) 50-75 years | 125 | 36.084 |  |  |
| Screening (AI) 40-75 years | 217 | 62.642 |  |  |
| Screening (AI) high risk population | 247 | 71.302 |  |  |
| Screening (AI) 50-75 years | 240 | 62.93 |  |  |
| Both eyes affected in OHT/POAG | 1 |  |  | Expert consultation |
| Both eyes affected in PACD | 0.64 | 0.0369 |  | [52] |
| **Diagnosis and Treatment costs** |  |  |  |  |
| **Diagnostics (Government facilities)** |  |  |  |  |
| Slit lamp examination | 58 | 16.743 | Gamma | [53] |
| Gonioscopy | 67 | 19.341 |  |  |
| Tonometry | 58 | 16.743 |  |  |
| Optical Coherence Tomography | 345 | 99.593 |  |  |
| Perimetry | 166 | 47.920 |  |  |
| **Diagnostics (Private facilities)** |  |  |  |  |
| Slit lamp examination | 500 | 144.338 | Gamma | [54], [55] |
| Gonioscopy | 450 | 129.904 |  |  |
| Tonometry | 100 | 28.868 |  |  |
| Optical Coherence Tomography | 3000 | 866.025 |  |  |
| Perimetry | 2000 | 577.350 |  | [54], [55], [56] |
| **Care seeking pattern (OPD)** |  |  |  |  |
| District Hospital | 0.113 | 0.0065 | Normal | [57], [58] |
| Government tertiary facility | 0.264 | 0.0153 |  |  |
| Private facilities | 0.623 | 0.0359 |  |  |
| **Care seeking pattern (IPD)** |  |  |  |  |
| District Hospital | 0.096 | 0.0055 | Normal | [57], [58] |
| Government tertiary facility | 0.224 | 0.0129 |  |  |
| Private facilities | 0.680 | 0.0393 |  |  |
| **% availability of medicines** |  |  |  |  |
| District Hospital | 0.25 | 0.0329 | Normal | [57] |
| Government tertiary facility | 0.18 | 0.0144 |  |  |
| **Treatment related costs (Government facilities)** |  |  |  |  |
| Consultation (District Hospital) | 258 | 74.478 | Gamma | [51] |
| Consultation (Government Tertiary Hospital) | 556 | 160.503 |  |  |
| Iridotomy- one eye | 8997 | 2597.210 |  |  |
| Trabeculectomy- one eye (District Hospital) | 12113 | 3496.722 |  |  |
| Trabeculectomy- one eye (Government Tertiary Hospital) | 17693 | 5107.529 |  |  |
| **Procurement prices (per month)** |  |  |  |  |
| Timolol | 5.36 | 1.547 | Gamma | [59] |
| Travoprost and timolol | 43.68 | 12.609 |  |  |
| Timolol and brimonidine | 25.74 | 7.430 |  |  |
| Dorzolamide | 38.08 | 10.993 |  |  |
| Travoprost | 106.38 | 30.709 |  |  |
| **Treatment related costs (Private facilities)** |  |  |  |  |
| Consultation (Private Hospital) | 1214 | 350.452 | Gamma | [57] |
| Iridotomy (one eye) | 45000 | 12990.381 |  | [60] |
| Trabeculectomy (one eye) | 35000 | 10103.630 |  | [61] |
| Timolol | 57 | 16.310 |  | [62] |
| Timolol and travoprost | 325 | 93.675 |  |  |
| Timolol, travoprost and Dorzolamide | 731 | 211.154 |  |  |
| Timolol, travoprost, Dorzolamide and brimonidine | 912 | 263.308 |  |  |
| **Direct non-medical costs** |  |  |  |  |
| OPD (Government facilities) | 64 | 18.475 | Gamma | [57] |
| OPD (Private facilities) | 94 | 27.135 |  |  |
| IPD (Government facilities) | 1075 | 310.326 |  |  |
| IPD (Private facilities) | 1440 | 415.692 |  |  |
| Screening | 50 | 14.434 |  | Expert Consultation |

# **Section S2. Details of costing of screening services**

We estimated the cost of implementation of screening for glaucoma at the Primary Health Centres in four considered models of screening: face-to-face screening in 40-75 years population, face-to-face screening in 40-75 years population at high risk of glaucoma, AI-supported screening in 40-75 years population, and AI-supported screening in 40-75 years population at high risk of glaucoma. The systematic screening has not been initiated at the Primary Health Centres. Therefore, a normative evaluation of costs was undertaken to assess the cost of screening, using empirical data on cost of primary healthcare services in India, as a part of studies undertaken and archived in the National Health System Cost Database of India [51].

For costing purpose, it was assumed that the screening would be offered to patients by optometrists, who would be supported by medical officers at primary health centres. Additionally, Community Health Officers (CHOs) at sub-centres and Accredited Social Health Activists (ASHAs) would be involved in social mobilization to access screening services at PHCs.

For face-to-face screening, we assumed that a room with medical officer and optometrist would be required. Additionally, registration room, and waiting area of the PHCs would be accessed by the patients attending the screening. In terms of equipment, Snellen’s chart, tonometer, non-mydriatic fundus camera, and pen torch would be used. There would be requirement of stationery and IEC material to implement the screening.

The space of rooms along with rental prices, stationary, and costs of utilities (electricity and water) for designated days were taken from the aforementioned published Indian costing studies [51]. The salaries of the involved staff were obtained through the National Health System Cost Database, and personal communication with government officials [35]. The cost of Snellen’s chart and non-mydriatic fundus camera were acquired from the National Health System Cost Database using the cost of health services study in India [40]. The life span of Snellen’s chart was considered to be 10 years, while that of non-mydriatic fundus camera, tonometer, and pen torch was 8 years. All the capital costs were annualized using a discount rate of 3%. The cost of IEC specific to glaucoma was obtained from financial estimation by state government in northern India. All the costs taken specifically from the published study were inflated from 2014-15 to 2022 using GDP deflator values. Apportioning of salaries was done according to time spent on the activity, while costs of other capital and non-capital items were apportioned according to a factor derived from expected number of patients to be screened within the catchment population and the total footfall at the centres.

Apart from these costs, it was considered necessary to include costs related to training of staff and supportive supervision to sustain the screening services. A one-day capacity building programme conducted by ophthalmologist and programme manager, was assumed for medical officers and optometrists to equip them with the skills required to conduct the screening, based on expert consultations. It was further assumed that medical officer would train the community health officers and ASHAs, so as to enable them to undertake social mobilization activities for uptake of screening services. The rental cost of a training room at district hospital, salaries of ophthalmologist and programme manager, quantity and cost of furniture, and training material, were acquired from the National Health System Cost Database using the estimates from ‘costing of health services study’. Further, incentives for training, provided to medical officer and optometrists were acquired from financial estimation by consultation with officials from state government in northern India. Apportioning of the resources used in training was done according to time. Further considering, that the staff of all PHCs (n=33) would be trained in a single training session at a district level facility, the cost of training per PHC was derived.

We considered six supervision visits by health authorities to the PHCs annually. The salaries of officials, medical officers, and optometrists, as well as the costs of space, furniture, and stationery used during supervision, were included in the screening costs.

In AI-supported screening, all costs assumed in the face-to-face screening were considered. The cost of software per scan was acquired through three rounds of consultations with the private suppliers. The suppliers provided the rate of software per scan, specific to the government. A mean of costs provided by all the suppliers was considered to calculate the unit cost of AI- supported screening.

The outputs of screening were computed at different coverage rates. Considering PHC on an average caters to 25000 people in its catchment area, and that 29.5% of population is in 40-75 years age group, and 17% in 15-75 years age group, we calculated the number of eligible populations for screening. Similarly, population at high risk of glaucoma was estimated using prevalence rates of hypertension, and diabetes, and population with family history of glaucoma. The number of people to screened were accordingly calculated at screening coverage rates of 25%, 50%, 65%, 75%, 85%, and 100%.

Table S2. Per person cost of screening according to variations in screening coverages (₹)

| Screening Coverage Rate | Face-to-face screening in 40-75 years age group | AI-supported screening in 40-75 years age group | Face-to-face screening in high-risk population | AI-supported screening in high-risk population | Face-to-face screening in 50-75 years age group | AI-supported screening in 50-75 years age group |
| --- | --- | --- | --- | --- | --- | --- |
| 25% | 151 | 266 | 227 | 342 | 208 | 323 |
| 50% | 112 | 227 | 150 | 265 | 140 | 255 |
| 65% | 102 | 217 | 132 | 247 | 125 | 240 |
| 75% | 98 | 213 | 124 | 239 | 118 | 233 |
| 85% | 95 | 210 | 118 | 233 | 112 | 227 |
| 100% | 91 | 206 | 111 | 226 | 106 | 221 |

**Break-up of costs for implementation of the glaucoma screening programme at 65% screening coverage rate**

Human resources and equipment contributed to the majority of the health systems costs to implement glaucoma screening programme (Figure S1). In face-to-face screening, human resources had a share of 69% in the overall costs and equipment contributed to 24% of the costs. However, the cost of equipment formed the major chunk of costs (65%) in the AI- supported screening.

Figure S1. Details of cost components in screening scenario

1. F2F screening

B. AI-supported screening

# **Section S3. Calculation of utility values in co-morbidities**

We derived the health state wise quality of life through face-to-face interviews with 297 patients diagnosed with glaucoma, using the EuroQoL Five-Dimensions Five-Levels (EQ-5D-5L) tool and the Indian value set. However, since the sample size of the study was not adequate to capture quality of life of glaucoma in presence of co-morbidities. Therefore, to calculate the combined utility values of glaucoma alongside the presence of hypertension, diabetes, or both conditions, were determined using the multiplicative method. The utility values associated with hypertension and/or diabetes were obtained from a previously published Indian study [50].

The formula used to derive the combined utility value was:

U _(A, B)_ = U_(nA, nB)_*(U_A_/U_nA_) *(U_B_/U_nB_)

Where, U _(A, B)_ is utility value in co-morbid condition (disease A, and disease B)

U_(nA, nB)_ is utility value in absence of both diseases A and B

U_A_ is utility value in disease A

U_B_ is utility value in disease B

U_nA_ is utility value in absence of disease A

U_nB_ is utility value in absence of disease B

To illustrate with an example: If A is hypertension, B is mild POAG,

U_(nA, nB)_ = 1, U_nA_ = 1, U_nB_ = 1, U_A_ = 0.89, U_B_= 0.8

The calculated utility value of hypertensive patients with OHT was 0.71

Table S3. Change in overall cost of care with varying availability of medicines at government-funded health facilities (₹ millions)

|  | No change in care seeking pattern | | | Improved coverage of care in public sector* | | | | | | | | | | | |
| --- | --- | --- | --- | --- | --- | --- | --- | --- | --- | --- | --- | --- | --- | --- | --- |
|  |  |  |  | Current drug availability | | | 50% drug availability | | | 75% drug availability | | | 100% availability | | |
|  | OOPE | HS | Total | OOPE | HS | Total | OOPE | HS | Total | OOPE | HS | Total | OOPE | HS | Total |
| High risk population (F2F) | 27 | 5 | 33 | 5 | 10 | 15 | 4 | 10 | 14 | 2 | 10 | 13 | 1 | 10 | 11 |
| High risk population (AI) | 28 | 7 | 35 | 6 | 12 | 17 | 4 | 12 | 16 | 3 | 12 | 14 | 1 | 12 | 13 |
| 40-75 years (F2F) | 32 | 6 | 39 | 6 | 12 | 18 | 4 | 12 | 16 | 3 | 12 | 15 | 1 | 12 | 13 |
| 40-75 years (AI) | 33 | 8 | 41 | 6 | 14 | 20 | 5 | 14 | 18 | 3 | 14 | 17 | 1 | 14 | 16 |

**^*^**^Considering 90% of the screened suspected glaucoma cases who seek confirmatory diagnosis and treatment at higher facilities, utilize government-funded facilities^

^OOPE: Out-of-pocket expenditure, HS: Health system costs, F2F: Face-to-face, AI: Artificial intelligence ₹: Indian National Rupee^

# **Section S4. Details of literature on economic evaluation of glaucoma screening**

Table S4. Evidence from existing literature on economic evaluation of glaucoma screening (1995-May 2024)

| S.No. | Author’s Name (year) | Type of glaucoma | Country / Perspective | Comparison Arms | Time Horizon | Discount Rate | WTP Threshold | Conclusion |
| --- | --- | --- | --- | --- | --- | --- | --- | --- |
| 1 | Anton et al (2023)  [63] | Open angle glaucoma | Spain/ Healthcare | 1. Open angle glaucoma screening (visual acuity, tonometry, OCT images, fundus photography) by optometrists and nurses  2. Opportunistic case finding | Lifetime | 3% | € 25,000/QALY | Implementing a glaucoma screening strategy in primary care settings using tonometry, fundus photographs, and OCT images for individuals over 55 years old is cost-effective in Spain with probability of 45%. |
| 2 | John et al (2017)  [64] | Primary open-angle glaucoma and primary angle closure disease | India/ Healthcare | 1.Community screening programme in urban India (refraction, direct ophthalmoscopy, IOP using tonopen, van herick test using slit lamp, perimetry)  2. No screening programme (with opportunistic case finding) | 10 years | 3% | ₹90884/QALY  GDP per capita (India 2014-2015) | Community glaucoma screening for 40-69-years olds in urban India is cost-effective. |
| 3 | Thomas et al (2015)  [65] | Not mentioned | Canada/ Healthcare and societal | 1. Teleglaucoma screening  2. In-person examination (No screening) | 30 years | 3% | $ 40,000/QALY | Tele glaucoma is cost-effective (100% probability) in comparison to no screening in rural Alberta. |
| 4 | John et al (2018)  [66] | Primary open-angle glaucoma and primary angle closure disease | India/ Healthcare | 1.Community screening programme in rural India (refraction, direct ophthalmoscopy, IOP using tonopen, van herick test using slit lamp, perimetry)  2. No screening programme (with opportunistic case finding) | 10 years | 3% | ₹ 120,739/QALY  GDP per capita (2016) | Community screening for glaucoma in rural India, compared to current practices, is cost-effective for the 40-69 years age group. |
| 5 | Vaahtoranta-Lehtonen et al (2007)  [67] | Not mentioned | Finland/ Healthcare | 1. Organized screening programme (IOP, fundus exam, autorefraction, visual field exam)  2. Opportunistic case finding | 20 years | 5% | Not mentioned | The probability of screening being cost-effective in 40-79 years age group at a frequency of once in 5 years was 29%. Organized glaucoma screening may be cost-effective in older age groups (65-79 years) at screening frequency of once in five years. |
| 6 | Xiao et al (2021)  [68] | Primary angle-closure disease | China/ Healthcare | 1. Glaucoma screening with artificial intelligence (AI) automated diagnosis  2. No screening (usual care) | 15 years | Costs: 5% | Not mentioned | Population screening with AI diagnosis reduced PACG progression risks in China. |
| 7 | Tang et al (2019)  [21] | Primary open-angle glaucoma (POAG) and Primary angle closure glaucoma (PACG) | China/ Societal | For PACG, POAG and combined (POAG and PACG) in rural and urban setting-  1. Community level screening (van herick test and optic nerve photographs)  2. Opportunistic case finding (No screening) | 30 years | 3.5% | Rural areas: $34.2  Urban areas: $558  3-times per-capita GDP | Combined screening for POAG and PACG in China is likely to be cost-effective. Screening for POAG (but not PACG) for individuals aged 50 years or older is not cost-effective in rural areas |
| 8 | Boivin et al (1996)  [69] | Primary open-angle glaucoma | Quebec (Canada)/ Healthcare | I. Screening with fundoscopy and tonometry  II. Tonometry alone.  Different screening scenarios-  1. Scenario 1- 3-yearly screening, ages 40-79  2. Scenario 2- Annual screening, ages 40-79  3. Scenario 3- 5-yearly screening, ages 40-79  4. Scenario 4 to 9-Varying rates affect blindness reduction and treatment.  5. Scenario 10- 3-yearly screening, ages 65-79  6. Scenario 11-  3-yearly tonometry screening, ages 40-79  7. Scenario 12- 3-yearly tonometry screening, ages 65-79 | Lifetime | Not mentioned | Not mentioned | Glaucoma screening is not cost-effective even when treatment efficacy is assumed to be as high as 50%. |
| 9 | Tuck et al (1997)  [70] | Primary open angle glaucoma, Chronic simple glaucoma (CSG), Normal pressure glaucoma (NPG) | UK/ Healthcare | 1. Ophthalmoscopy  2. Tonometry  3. Perimetry  (screening was conducted by ophthalmologists or optometrists) | 6 years | Not mentioned | Not mentioned | The most cost-effective screening combines routine ophthalmoscopy and tonometry, with selective perimetry. |
| 10 | Burr et al (2014)  [71] | Open-angle glaucoma | UK/ Healthcare | 1. Screening examination by glaucoma trained optometrist (IOP, assessment of the optic nerve and visual field test)  2. Screening examination by technician (IOP, automated assessment of the optic nerve and visual fields)  3. Current practice | Lifetime | 3.5% | £30,000/QALY | Screening is not cost effective compared to current practice. Screening is most likely to be cost-effective for people who have a family history of OAG and people of black ethnicity, with a 10-year screening interval |
| 11 | Liu et al (2023)  [72] | Primary open-angle glaucoma | UK and Australia/ Payer | 1. Genetic screening using a polygenic risk score (PRS)  2. Comprehensive optometrists review every 2 years for those identified as high risk based on their PRS (current POAG screening) | Lifetime | UK: 3.5%  Australia: 5% | UK: £20,000 to £30,000  Australia: $54,808 | Genetic screening using a polygenic risk score for glaucoma is likely cost-effective in Australia and potentially in the UK with 80% probability at the given threshold. |
| 12 | Rein et al (2009)  [73] | Primary open angle glaucoma | USA/ Healthcare | 1. Assessment and treatment according to EMGT (opportunistic assessment- IOP, automated VF tests, gonioscopy with conservative treatment)  2. Assessment and treatment according to CIGTS (opportunistic assessment with optimistic treatment)  3. No assessment and no treatment | Lifetime | 3% | $ 28,000/QALY gained  1 times U.S. GDP per capita | Glaucoma treatment is cost-effective, particularly when excluding diagnostic assessment costs or assuming optimistic treatment efficacy. |
| 13 | Hernandez et al  [72] | Primary open angle glaucoma | UK/ Healthcare | 1. Screening examination by glaucoma trained optometrist (IOP, assessment of the optic nerve and visual field test)  2. Screening examination by technician (IOP, automated assessment of the optic nerve and visual fields)  3. Current practice (opportunistic identification by community optometrists as routine test) | Lifetime | 3.5% | £30,000 per QALY gained | For a 40-year-olds cohort a ‘technician’ screening strategy compared with current practice has an ICER below the threshold when prevalence is approximately 10%. No age cohort and no prevalence level in screening by the glaucoma optometrist instead of screening by the technician was cost-effective. Targeted screening of 40- to 50-year-olds with a risk factor, (e.g. black ethnicity or those with a family history of glaucoma), is more likely to be cost-effective assuming a prevalence of OAG between 3% to 4% and a screening interval of ten years. |

# **Section S5. Compliance to CHEERS Checklist**

Table S5. CHEERS Checklist

|  | **Item** | **Guidance for Reporting** | **Reported in section** |
| --- | --- | --- | --- |
| **TITLE** | | |  |
| Title | 1 | Identify the study as an economic evaluation and specify the interventions being compared. | Page 1 |
| **ABSTRACT** | | |  |
| Abstract | 2 | Provide a structured summary that highlights context, key methods, results and alternative analyses. | Page 1 |
| **INTRODUCTION** | | |  |
| Background and objectives | 3 | Give the context for the study, the study question and its practical relevance for decision making in policy or practice. | Page 3, 4 |
| **METHODS** | | |  |
| Health economic  analysis plan | 4 | Indicate whether a health economic analysis plan was developed and where available. |  |
| Study population | 5 | Describe characteristics of the study population (such as age range, demographics, socioeconomic, or clinical characteristics). | Page 4,5 |
| Setting and location | 6 | Provide relevant contextual information that may influence findings. | Page 4,5 |
| Comparators | 7 | Describe the interventions or strategies being compared and why chosen. | Page 4,5 |
| Perspective | 8 | State the perspective(s) adopted by the study and why chosen. | Page 5 |
| Time horizon | 9 | State the time horizon for the study and why appropriate. | Page 3 |
| Discount rate | 10 | Report the discount rate(s) and reason chosen. | Page 5 |
| Selection of outcomes | 11 | Describe what outcomes were used as the measure(s) of benefit(s) and harm(s). | Page 5 |
| Measurement of outcomes | 12 | Describe how outcomes used to capture benefit(s) and harm(s) were measured. | Page 8 |
| Valuation of outcomes | 13 | Describe the population and methods used to measure and value outcomes. | Page 4,5,8 |
| Measurement and valuation of resources  and costs | 14 | Describe how costs were valued. | Page 7,8 |
| Currency, price date, and conversion | 15 | Report the dates of the estimated resource quantities and unit costs, plus the currency and year of conversion. | Page 5,7,8 |
| Rationale and  description of model | 16 | If modelling is used, describe in detail and why used. Report if the model is publicly available and where it can be accessed. | Page 5 |
| Analytics and assumptions | 17 | Describe any methods for analysing or statistically transforming data, any extrapolation methods, and approaches for validating any model used. | Page 8, 14 |
| Characterizing heterogeneity | 18 | Describe any methods used for estimating how the results of the study vary for sub-groups. | Page 4 |
| Characterizing  distributional effects | 19 | Describe how impacts are distributed across different individuals or adjustments made to reflect priority populations. |  |
| Characterizing uncertainty | 20 | Describe methods to characterize any sources of uncertainty in the analysis. | Page 8, 9 |
| Approach to engagement with patients and others affected by the study | 21 | Describe any approaches to engage patients or service recipients, the general public, communities, or stakeholders (e.g., clinicians or payers) in the design of the study. | Page 4 |
| **RESULTS** | | |  |
| Study parameters | 22 | Report all analytic inputs (e.g., values, ranges, references) including uncertainty or distributional assumptions. | Page 2-7 of supplement |
| Summary of main results | 23 | Report the mean values for the main categories of costs and outcomes of interest and summarise them in the most appropriate overall measure. | Page 9-10 |
| Effect of uncertainty | 24 | Describe how uncertainty about analytic judgments, inputs, or projections affect findings. Report the effect of choice of discount rate and time horizon, if applicable. | Page 10-11 |
| Effect of engagement with patients and others affected by the study | 25 | Report on any difference patient/service recipient, general public, community, or stakeholder involvement made to the approach or findings of the study |  |
| **DISCUSSION** | | |  |
| Study findings, limitations, generalizability, and current knowledge | 26 | Report key findings, limitations, ethical or equity considerations not captured, and how these could impact patients, policy, or practice. | Page 11-14 |
| **OTHER RELEVANT INFORMATION** | | | |
| Source of funding | 27 | Describe how the study was funded and any role of the funder in the identification, design, conduct, and reporting of the analysis | Page 15 |
| Conflicts of interest | 28 | Report authors conflicts of interest according to journal or  International Committee of Medical Journal Editors requirements. | Page 15 |

**References**

1. Dandona L, Dandona R, Srinivas M, Mandal P, John RK, McCarty CA, Rao GN. Open-angle glaucoma in an urban population in southern india: The andhra pradesh eye disease study.2000;107(9):1702–1709.
2. Dandona L, Dandona R, Mandal P, Srinivas M, John RK, McCarty CA, Rao GN. Angle-closure glaucoma in an urban population in southern india: The andhra pradesh eye disease study. 2000;107(9):1710–1716.
3. Rajendrababu S, Bansal O, Shroff S, Senthilkumar VA, Uduman MS. Visual field-based grading of disease severity in newly diagnosed primary open angle glaucoma patients presenting to a tertiary eye care centre in India. Int Ophthalmol. 2021 Sep;41(9):3135-3143.
4. Kulkarni U. Early Detection of Primary Open Angle Glaucoma: Is It Happening? Journal of Clinical and Diagnostic Research. 2012;6(4):667-670
5. Suram V, Shaikh S, Chadaram B. Clinical profile of the stages of primary angle closure disease in a tertiary eye hospital in South India. International Journal of Pharmaceutical and Medical Research. 2015;3(4):11-15
6. Garudadri C, Senthil S, Khanna RC, Sannapaneni K, Rao HBL. *Prevalence and Risk Factors for Primary Glaucomas in Adult Urban and Rural Populations in the Andhra Pradesh Eye Disease Study. 2010;117(7):1352–1359.*
7. Sharfuddin Ahmed M, Ullah AY, Barman N, et al. Risk factors associated with elevated intraocular pressure: a population-based study in a rural community of Bangladesh. *BMJ Open Ophthalmol*. 2023;8(1):e001386. doi:10.1136/bmjophth-2023-001386
8. Nislawati R, Taufik Fadillah Zainal A, Ismail A, Waspodo N, Kasim F, Gunawan AMAK. Role of hypertension as a risk factor for open-angle glaucoma: a systematic review and meta-analysis. BMJ Open Ophthalmol. 2021;6(1):e000798
9. Burr JM, Mowatt G, Hernandez R, Siddiqui MAR, Cook J, Lourenco T, Ramsay C, Vale L, Fraser C, Azuara-Blanco A, Deeks J, Cairns J, Wormald R, McPherson S, Rabindranath K, Grant A. The clinical effectiveness and cost-effectiveness of screening for open angle glaucoma: a systematic review and economic evaluation. 2007;11(41).
10. Vijaya L, George R, Arvind H, et al. Prevalence of primary angle-closure disease in an urban south Indian population and comparison with a rural population. The Chennai Glaucoma Study. *Ophthalmology*. 2008;115(4):655-660.e1. doi:10.1016/j.ophtha.2007.05.034
11. Paul C, Sengupta S, Banerjee S, Choudhury S. Angle closure glaucoma in rural and urban populations in eastern India-The Hooghly River Glaucoma Study. Indian J Ophthalmol. 2018;66(9):1285-1290.
12. Zhang Y, Zhang Q, Thomas R, Li SZ, Wang NL. Development of angle closure and associated risk factors: The Handan eye study. Acta Ophthalmol. 2022;100(1):e253-e261.
13. Vijaya L, Rashima A, Panday M, Choudhari NS, Ramesh SV, Lokapavani V, Boddupalli SD, Sunil GT, George R. *Predictors for Incidence of Primary Open-Angle Glaucoma in a South Indian Population. Ophthalmology. 2014;121(7):1370–1376.*
14. Choudhari NS, Khanna RC, Marmamula S, Mettla AL, Giridhar P, Banerjee S, Shekhar K, Chakrabarti S, Murthy GVS, Gilbert C, Rao GN; Andhra Pradesh Eye Disease Study Group. Fifteen-Year Incidence Rate of Primary Angle Closure Disease in the Andhra Pradesh Eye Disease Study. Am J Ophthalmol. 2021;229:34-44.
15. Ramakrishnan S, Zachariah G, Gupta K, Shivkumar Rao J, Mohanan PP, Venugopal K, Sateesh S, Sethi R, Jain D, Bardolei N, Mani K, Kakar TS, Kidambi B, Bhushan S, Verma SK, Bhargava B, Roy A, Kothari SS, Gupta R, Bansal S, Sood S, Nath RK, Tyagi S, Gupta MD, Girish MP, Kalra IPS, Wander GS, Gupta S, Mandal S, Senguttuvan NB, Subramanyam G, Roy D, Datta S, Ganguly K, Routray SN, Mishra SS, Singh BP, Bharti BB, Das MK, Kumar S, Goswami KC, Bahl VK, Chandra S, Banerjee A, Guha S, Deb PK, Chopra HK, Deedwania P, Seth A; CSI-Great India BP Campaign Investigators. Prevalence of hypertension among Indian adults: Results from the great India blood pressure survey. Indian Heart J. 2019;71(4):309-313.
16. Anjana RM, Pradeepa R, Deepa M, et al. Prevalence of diabetes and prediabetes (impaired fasting glucose and/or impaired glucose tolerance) in urban and rural India: phase I results of the Indian Council of Medical Research-INdia DIABetes (ICMR-INDIAB) study. *Diabetologia*. 2011;54(12):3022-3027. doi:10.1007/s00125-011-2291-5
17. Prabhakaran D, Jeemon P, Ghosh S, et al. Prevalence and incidence of hypertension: Results from a representative cohort of over 16,000 adults in three cities of South Asia. *Indian Heart J*. 2017;69(4):434-441. doi:10.1016/j.ihj.2017.05.021
18. Mohan V, Deepa M, Anjana RM, Lanthorn H, Deepa R. Incidence of diabetes and pre-diabetes in a selected urban south Indian population (CUPS-19). J Assoc Physicians India. 2008;56:152-7.
19. Panday M, George R, Asokan R, et al. Six-year incidence of ocular hypertension in a South Indian population: the Chennai eye disease incidence study. *Br J Ophthalmol*. 2015;99(5):604-608. doi:10.1136/bjophthalmol-2014-305714
20. Kass MA, Heuer DK, Higginbotham EJ, et al. The Ocular Hypertension Treatment Study: a randomized trial determines that topical ocular hypotensive medication delays or prevents the onset of primary open-angle glaucoma. *Arch Ophthalmol*. 2002;120(6):701-830. doi:10.1001/archopht.120.6.701
21. Tang J, Liang Y, O'Neill C, Kee F, Jiang J, Congdon N. Cost-effectiveness and cost-utility of population-based glaucoma screening in China: a decision-analytic Markov model. Lancet Glob Health. 2019;7(7):e968-e978.
22. Burr JM, Mowatt G, Hernández R, et al. The clinical effectiveness and cost-effectiveness of screening for open angle glaucoma: a systematic review and economic evaluation. *Health Technol Assess*. 2007;11(41):iii-190. doi:10.3310/hta11410
23. Heijl A, Leske MC, Bengtsson B, Hyman L, Bengtsson B, Hussein M; Early Manifest Glaucoma Trial Group. Reduction of intraocular pressure and glaucoma progression: results from the Early Manifest Glaucoma Trial. Arch Ophthalmol. 2002 Oct;120(10):1268-79.
24. Ordóñez JE, Ordóñez A, Osorio UM. Cost-effectiveness analysis of iStent trabecular micro-bypass stent for patients with open-angle glaucoma in Colombia. *Curr Med Res Opin*. 2019;35(2):329-340. doi:10.1080/03007995.2018.1506022
25. Pantalon A, Feraru C, Tarcoveanu F, Chiselita D. Success of Primary Trabeculectomy in Advanced Open Angle Glaucoma. *Clin Ophthalmol*. 2021;15:2219-2229. Published 2021 May 27. doi:10.2147/OPTH.S308228
26. Yuasa Y, Sugimoto Y, Hirooka K, et al. Effectiveness of trabeculectomy with mitomycin C for glaucomatous eyes with low intraocular pressure on treatment eye drops. *Acta Ophthalmol*. 2020;98(1):e81-e87. doi:10.1111/aos.14195
27. Hattenhauer MG, Johnson DH, Ing HH, et al. The probability of blindness from open-angle glaucoma. *Ophthalmology*. 1998;105(11):2099-2104. doi:10.1016/S0161-6420(98)91133-2
28. Thomas R, Parikh R, Muliyil J, Kumar RS. Five-year risk of progression of primary angle closure to primary angle closure glaucoma: a population-based study. Acta Ophthalmol Scand. 2003;81(5):480-5.
29. George R, Ve RS, Vijaya L. Glaucoma in India: estimated burden of disease. *J Glaucoma*. 2010;19(6):391-397. doi:10.1097/IJG.0b013e3181c4ac5b
30. Sihota R. An Indian perspective on primary angle closure and glaucoma. Indian J Ophthalmol. 2011;59 Suppl(Suppl1):S76-81.
31. Yip JL, Foster PJ, Uranchimeg D, Javzandulam B, Javzansuren D, Munhzaya T, Lee PS, Baassanhuu J, Gilbert CE, Khaw PT, Johnson GJ, Nolan WP. Randomised controlled trial of screening and prophylactic treatment to prevent primary angle closure glaucoma. Br J Ophthalmol. 2010;94(11):1472-7.
32. Yuan Y, Wang W, Xiong R, Zhang J, Li C, Yang S, Friedman DS, Foster PJ, He M. Fourteen-Year Outcome of Angle-Closure Prevention with Laser Iridotomy in the Zhongshan Angle-Closure Prevention Study: Extended Follow-up of a Randomized Controlled Trial. Ophthalmology. 2023;130(8):786-794.
33. Quek DTL, Koh VT, Tan GS, Perera SA, Wong TT, Aung T. Blindness and long-term progression of visual field defects in chinese patients with primary angle-closure glaucoma. Am J Ophthalmol. 2011;152(3):463-469.
34. Government of India. Sample Registration System (SRS) abridged Life Table 2014-18: Office of the Registrar General & Census Commissioner, India; Available from: <https://censusindia.gov.in/nada/index.php/catalog/42517>, last accessed on 22.04.2024.
35. Rachapelle S, Legood R, Alavi Y, et al. The cost-utility of telemedicine to screen for diabetic retinopathy in India. Ophthalmology. 2013;120(3):566-573.
36. Waisbourd M, Bond EA, Sullivan T, Hu WD, Shah SB, Molineaux JS, Harjeet S, George L, Myers JS, Hark LA, Katz LJ. *Evaluation of Nonmydriatic Hand-held Optic Disc Photography Grading in the Philadelphia Glaucoma Detection and Treatment Project. Journal of Glaucoma. 2016;25(5):e520–e525.*
37. Nagarajan S, Velayutham V, Ezhumalai G. Comparative evaluation of applanation and indentation tonometers in a community ophthalmology setting in Southern India. Saudi J Ophthalmol. 2016;30(2):83-7. doi: 10.1016/j.sjopt.2015.11.002.
38. Chou R, Selph S, Blazina I, et al. *Screening for Glaucoma in Adults: A Systematic Review for the U.S. Preventive Services Task Force*. Rockville (MD): Agency for Healthcare Research and Quality (US); May 2022.
39. Thomas R, George T, Braganza A, Muliyil J. The flashlight test and van Herick's test are poor predictors for occludable angles. *Aust N Z J Ophthalmol*. 1996;24(3):251-256. doi:10.1111/j.1442-9071.1996.tb01588.x
40. Rao DP, Shroff S, Savoy FM, S S, Hsu CK, Negiloni K, Pradhan ZS, P V J, Sivaraman A, Rao HL. Evaluation of an offline, artificial intelligence system for referable glaucoma screening using a smartphone-based fundus camera: a prospective study. Eye (Lond). 2024 Apr;38(6):1104-1111. doi: 10.1038/s41433-023-02826-z. Epub 2023 Dec 13. PMID: 38092938; PMCID: PMC11009383.
41. Ramakrishnan R, Abdul Khadar SM, Srinivasan K, Kumar H, Vijayakumar V. Diabetes mellitus in the Tamil Nadu State-Noncommunicable diseases nurse model in diabetic retinopathy screening. Indian J Ophthalmol. 2020;68(Suppl 1):S78-S82.
42. Nirmalan PK, Katz J, Robin AL, Krishnadas R, Ramakrishnan R, Thulasiraj RD, Tielsch J. Utilisation of eye care services in rural south India: the Aravind Comprehensive Eye Survey. Br J Ophthalmol. 2004;88(10):1237-41.
43. Buttan S, Gascoyne B, Das S, Schmidt E. Piloting targeted glaucoma screening: experiences of eye care services in Ganjam district, Odisha state, India. Int Health. 2022;14(Suppl 1):i29-i36.
44. Rengappa R, Chandrashekharan S, Gunaselvi R, Maheshwari D, Kader MA, Chakrabarty S. Agreement of findings of glaucoma screening between trained vision center technicians and glaucoma specialists at a tertiary hospital in South India. Indian J Ophthalmol. 2021;69(4):871-875.
45. Mohanty SK, Mishra ARS, Upadhyay AK, O’Donnell O, Maurer SK. Sociodemographic and geographic inequalities in diagnosis and treatment of older adults’ chronic conditions in India: a nationally representative population-based study. BMC Health Serv Res. 2023;3:332.
46. Vijaya L, George R, Baskaran M, et al. Prevalence of primary open-angle glaucoma in an urban south Indian population and comparison with a rural population. The Chennai Glaucoma Study. *Ophthalmology*. 2008;115(4):648-654.e1. doi:10.1016/j.ophtha.2007.04.062
47. Rajurkar K, Dubey S, Gupta PP, John D, Chauhan L. Compliance to topical anti-glaucoma medications among patients at a tertiary hospital in North India. *J Curr Ophthalmol*. 2018;30(2):125-129. Published 2018 Jun 8. doi:10.1016/j.joco.2017.09.002
48. Sharma D, Prinja S, Aggarwal AK, Rajsekar K, Bahuguna P. Development of the Indian Reference Case for undertaking economic evaluation for health technology assessment. *Lancet Reg Health Southeast Asia*. 2023;16:100241. Published 2023 Jun 17. doi:10.1016/j.lansea.2023.100241
49. Brar S, Kaur G, Muniyandi M, Karikalan N, Bano H, Bhansali A, Jain S, Kumari S, Prinja S. Cost of screening, out-of-pocket expenditure & quality of life for diabetes & hypertension in India. Indian J Med Res. 2023 Jun;157(6):498-508. doi: 10.4103/ijmr.IJMR_389_20. PMID: 37530305; PMCID: PMC10466497.
50. Purohit N, Goyal A, Gupta PC, Thattaruthody, Prinja S. Assessing quality of life in patients with cataract and glaucoma. Indian Journal of Ophthalmology. 2024. Accepted for publication.
51. National Health System Cost Database from India. Available from: <https://www.healtheconomics.pgisph.in/costing_web/index.php?action=gen_output>, last accessed on 22.10.2023
52. Sihota R. An Indian perspective on primary angle closure and glaucoma. *Indian J Ophthalmol*. 2011;59 Suppl(Suppl1):S76-S81. doi:10.4103/0301-4738.73687
53. Ministry of Health and Family Welfare. Government of India. Central Government Health Scheme rates. New Delhi. 2021. Available from: <https://cghs.gov.in/CghsGovIn/faces/ViewPage.xhtml>, last accessed on 22.10.2023
54. Medanta- The Medicity. Schedule of charges. 2018. Available from: <https://ptcul.org/document/medanta-list-2285-26-12-2018.pdf>
55. Lybrate. Glaucoma surgery cost in India. Available from: https://www.lybrate.com/cost/doctors-for-glaucoma-surgery/procedure
56. Eye Mantra. Glaucoma Visual Field Test: Know All about it. Available from: <https://eyemantra.in/glaucoma/visual-field-tests/>
57. Ministry of Statistics & Programme Implementation, Government of India. India - Household Social Consumption: Health, NSS 75th Round Schedule-25.0: July 2017-June 2018. Available from: <http://microdata.gov.in/nada43/index.php/catalog/152>, last accessed on 22.10.2023
58. NSSO. Health in India NSSO 71st Round. New Delhi: Ministry of Statistics and Programme Implementation; 2016. Contract No.: Report No. 574 (71/25.0).
59. *Drugs, Surgical and Sutures*. [http://www.rmsc.health.rajasthan.gov.in/content/raj/medical/rajasthan-medical-services-corporation-ltd-/en/Approved-Rate-Lists/DrugsRC.html#](http://www.rmsc.health.rajasthan.gov.in/content/raj/medical/rajasthan-medical-services-corporation-ltd-/en/Approved-Rate-Lists/DrugsRC.html)
60. Hexa Health. Iridotomy cost in India. Available from: <https://www.hexahealth.com/treatment/iridotomy-cost>
61. Pristyn Care. Glaucoma Surgery Cost in India. Available from: https://www.pristyncare.com/cost/glaucoma/#:~:text=Filtering%20Surgery%20or%20Trabeculectomy&text=This%20opening%20allows%20the%20fluid,40%2C000%20approximately.
62. Banga HK, Gupta AK, Singh G. Volumetric and cost evaluation study of glaucoma medical therapy. Int J Appl Basic Med Res. 2015;5(2):96-9.
63. Anton A, Serrano D, Nolivos K, Fatti G, Zmuc N, Crespo C, Monleon-Getino T, Sotelo K, Morilla A, García V, Comas M, Castells X. Cost-Effectiveness of Screening for Open Angle Glaucoma Compared With Opportunistic Case Finding. J Glaucoma. 2023 Feb 1;32(2):72-79. doi: 10.1097/IJG.0000000000002132. Epub 2022 Oct 3. PMID: 36696355.
64. John D, Parikh R. Cost-effectiveness and cost utility of community screening for glaucoma in urban India. Public Health. 2017 Jul;148:37-48. doi: 10.1016/j.puhe.2017.02.016. Epub 2017 Apr 6. PMID: 28404532.
65. Thomas S, Hodge W, Malvankar-Mehta M. The Cost-Effectiveness Analysis of Teleglaucoma Screening Device. PLoS One. 2015 Sep 18;10(9):e0137913. doi: 10.1371/journal.pone.0137913. PMID: 26382956; PMCID: PMC4575061.
66. John D, Parikh R. Cost-effectiveness of community screening for glaucoma in rural India: a decision analytical model. Public Health. 2018 Feb;155:142-151. doi: 10.1016/j.puhe.2017.11.004. Epub 2018 Feb 2. PMID: 29407529.
67. Vaahtoranta-Lehtonen H, Tuulonen A, Aronen P, et al. Cost effectiveness and cost utility of an organized screening programme for glaucoma. Acta Ophthalmol Scand 2007; 85: 508–18.
68. Xiao X, Xue L, Ye L, Li H, He Y. Health care cost and benefits of artificial intelligence-assisted population-based glaucoma screening for the elderly in remote areas of China: a cost-offset analysis. BMC Public Health. 2021 Jun 4;21(1):1065.

Boivin JF. Cost effectiveness of screening for primary open angle glaucoma. J Med Screen. 1996;3(3):154–63.

1. Tuck MW, Crick RP. The cost-effectiveness of various modes of screening for primary open angle glaucoma. Ophthalmic Epidemiol. 1997;4(1):3–17.
2. Burr JM, Mowatt G, Hernandez R, et al. The clinical effectiveness and cost-effectiveness of screening for open angle glaucoma: a systematic review and economic evaluation. Health Technol Assess 2007; 11: 1–190
3. Liu Q, Davis J, Han X, Mackey DA, MacGregor S, Craig JE, Si L, Hewitt AW. Cost-effectiveness of polygenic risk profiling for primary open-angle glaucoma in the United Kingdom and Australia. Eye (Lond). 2023 Aug;37(11):2335-2343. doi: 10.1038/s41433-022-02346-2. Epub 2022 Dec 13. PMID: 36513856; PMCID: PMC10366078.
4. Rein DB, Wittenborn JS, Lee PP, Wirth KE, Sorensen SW, Hoerger TJ, Saaddine JB. The cost-effectiveness of routine office-based identification and subsequent medical treatment of primary open-angle glaucoma in the United States. Ophthalmology. 2009 May;116(5):823-32. doi: 10.1016/j.ophtha.2008.12.056. Epub 2009 Mar 14. PMID: 19285730.
5. Hernandez R, Burr JM, Vale L. Economic evaluation of screening for open angle glaucoma. International Journal of Technology Assessment in Healthcare. 2008;24:204-11.
