# Supplementary material for: Seizing the silent vision loss: cost–utility analysis of population-based glaucoma screening in India
Source: BMJ Open. 2025 Apr 3;15(4):e098113. doi: 10.1136/bmjopen-2024-098113 (PMC11969579; doi:10.1136/bmjopen-2024-098113)
Supplement: Supplementary file 2 [file bmjopen-2024-098113.reviewer_comments.pdf]

PEER REVIEW HISTORY

BMJ Open publishes all reviews undertaken for accepted manuscripts. Reviewers are asked to complete a checklist review form and are provided with free text boxes to elaborate on their assessment. These free text comments are reproduced below.

ARTICLE DETAILS

Title (Provisional)

Seizing the silent vision loss: Cost-Utility Analysis of Population-Based Glaucoma Screening in India

Authors

Purohit, Neha; Buttan, Sandeep; Gupta, Parul Chawla; Choudhury, Ranjan Kumar; Soundappan, Kathirvel; Kotwal, Atul; Prinja, Shankar

---

VERSION 1 - REVIEW

**Reviewer**	**1**
**Name**	**Dack, Kyle**
**Affiliation**	**University of Bristol, MRC Integrative Epidemiology Unit**
**Date**	**20-Feb-2025**
**COI**	**None**

This study simulates 12 glaucoma screening programmes, and evaluates their cost-utility. I have experience with simulated data and cost-utility evaluations, but having read the paper several times I find it hard to follow the methods and results. Particularly the methods are quite mixed up, making it hard to follow how the data was generated, and what comparisons were performed. This makes it hard to follow the results – in certain places I am unsure whether the results are descriptive or if inference testing is performed. Several questionnaire items I have marked as "NA" because I currently cannot determine them due to lack of information.

Further major concerns are that (1) it is unclear from the title/abstract that this uses simulated data. (2) There are potentially issues regarding multiple testing (12 screening strategies, 5 primary outcomes) inflating the risk of spurious results. (3)Population characteristics such as sex and socioeconomic status are not considered and the generalizability of the results should be discussed more.

Abstract

1. Line 30. It would be clearer to replace "considered" with "included" or similar.

2. The "primary and secondary outcomes" subheading only lists primary outcomes, of which there are 5, multiplied by 12 screening strategies. If all of these are indeed primary outcomes, it sounds as if there are 60 primary comparisons? This significantly increases the risk of type 1 error due to multiple testing, i.e. the risk of spurious results. The authors may wish to select fewer primary outcomes, or apply statistical correction for multiple testing.

3. Line 51-53. The results are quite vague – it would be more useful to state the precise screening programme. Point estimates should also include an uncertainty measure ,e.g. the 95% CI.

Introduction

4. The introduction is clear and well written. However, it is not really explained why quality-adjusted life years seems to be the headline comparison (this is my assumption, based on this outcome being the first result reported in the abstract). What is the relevance of this measure, compared to say, risk of blindness?

The final paragraph states that the aim is to compare cost-utility – again I am unsure how that links to two of the primary outcomes: "life years, quality adjusted life years"

Methods

6. The beginning of the methods describes the development of the interventions, with 4 target populations x 2 screening methods x 2 screening frequencies. There are 16 possible combinations which result in 12 selected interventions. It would be helpful early in the methods to directly state (a list or table) which screening strategies were selected. It is also not clear which 4 possible combinations were dropped and why.

7. It is only clear to me from page 5 line 37 that this is a simulation study and does not use patient data. This should be more clearly signposted in the abstract and introduction, because it is not directly stated.

8. In light of the above I suggest reorganizing the methods. It is currently a bit muddled – the method to compare screening strategies is described before we know how the data was generated, or what the model includes. It will be easier to follow if it began by describing how the data was simulated – model parameters, outcomes, costs, and the 4 target populations. Followed by explaining how this was put together in a single model, including a list of all variables. Then describing the comparisons and statistical tests performed, and finally the sensitivity analyses.

9. Having read the methods several times, some important aspects are quite unclear to me.

a. how the base populations are generated. There are 4 target populations, differing by age and risk status, but it is hard to follow what parameters apply to each population and precisely how they were generated – I do not think I could replicate these populations given the current information.

b. The total list of variables included in the model. E.g, it is unclear whether aspects such as sex or socioeconomic status are considered – it is not stated so I assume not?

c. The total list of comparisons. From the abstract I assume there are 60, due to 12 interventions x 5 primary outcomes, but a concise explanation of the comparisons of interest is currently missing from the methods to confirm this.

Results

10. It would be useful to begin the results by describing the simple characteristics of the 4 target populations.

11. Table 1. The statistical method should be included in the table heading. Brackets are not explained. I don't think 4 decimal places is necessary and it harms the readability of this table, if reduced to 2dp it may be possible to fit all data on a single row. The cost columns are missing units (there is a symbol but not rendered). It is not clear why "40-70 years populations" are below the "50-70 years" subheading.

12. Table 2. Please add the statistical method. Are any uncertainty measures reported? I am unable to tell because it is not clear which method was used to create these percentages.

13. Throughout the paper various percentages are reported but again I am unsure regarding the certainty of these, and whether certainty estimates are generated, but if available they should be reported.

Discussion

14. A limitation which I did not see discussed is that the target populations seem to be treated as homogenous, but there could be variation due to sex, socioeconomic status, morbidity, which does not seem to be estimated here. So I wonder if this impacts the generalizability – who do these results apply to?

**Reviewer**	**2**
**Name**	**Olawoye, Olusola**
**Affiliation**	**University College Hospital, Department of Ophthalmology**
**Date**	**23-Feb-2025**
**COI**	**None**

I congratulate the authors of this study. They have taken time to be as detailed and comprehensive as possible and the study provides information about the cost effectiveness of population screening for glaucoma in India. I have a few comments.

1. The authors mentioned in their methodology that "The current scenario is that of opportunistic diagnosis by the ophthalmologists at higher level of care. However what the

authors are considering here is population screening for glaucoma . The diagnosis, can take place either at secondary or tertiary eye care. The authors seem to confuse screening with diagnosis. They seem to compare population-based glaucoma screening strategies with opportunistic glaucoma diagnosis, which is performed by ophthalmologists. Page 5 lines 5-7.

2. The authors compared opportunistic diagnosis with population screening. It would seem that both might not be cost-effective. Did the authors evaluate whether opportunistic diagnosis is cost-effective? Comparing these two (population screening and opportunistic diagnosis) would seem to mean that opportunistic diagnosis is cost-effective.

3. The authors said that their study was similar to studies in high-income countries; however these studies are entirely different as the study by Burr et al compared population screening with no screening at all, but this study is comparing population screening with opportunistic diagnosis at tertiary level of care

4. The authors referred to a study in the United States where opportunistic diagnosis of glaucoma during routine ophthalmologic examinations is considered cost-effective relative to scenarios involving no diagnosis or treatment. This baseline is fundamental as it gives a yardstick to understand the comparison. The authors might have done this first before comparing opportunistic diagnosis to population study

5. Some of the references are wrong. The authors need to go over all the references. The references are not correct. Kindly check references 8 and 9 which the authors said were studies done in Asia. The current references there do not match some of the statements

6. The authors did not mention anything about health insurance. It would be interesting to know how this affects the cost-effectiveness of screening and out-of-pocket payments.

7. The authors mentioned that patients with hypertension and diabetes are at high risk for glaucoma using some studies conducted in India. These medical diseases in themselves potentially increase the rate of blindness from glaucoma in patients with them. It is hoped that the authors did not factor in the treatment of these other co-existing diseases

8. The authors may consider rephrasing their conclusions to sort of reflect this: Population screening using the current glaucoma care pathways, where most persons are seen in private facilities in India, is not cost-effective. To make it cost-effective, the government must provide some financing for diagnosis and treatment, and medications must be available.

Well done to the authors

VERSION 1 - AUTHOR RESPONSE

Reviewer: 1

Dr. Kyle Dack, University of Bristol
Comments to the Author:

This study simulates 12 glaucoma screening programmes and evaluates their cost-utility. I have experience with simulated data and cost-utility evaluations but having read the paper several times I find it hard to follow the methods and results. Particularly the methods are quite mixed up, making it hard to follow how the data was generated, and what comparisons were performed. This makes it hard to follow the results – in certain places I am unsure whether the results are descriptive or if inference testing is performed. Several questionnaire items I have marked as "NA" because I currently cannot determine them due to lack of information.

Further major concerns are that:

(1) it is unclear from the title/abstract that this uses simulated data.

We thank the reviewer for the comment. As rightly understood by the reviewer, the study relies on data simulations to assess the cost-utility of glaucoma screening models, compared to current practice in India. This is outlined under the 'Design' sub-heading in the abstract on page 1:

'Design: Cost-utility analysis using mathematical model comprising of decision tree and Markov model'

This gives an early indication that the analysis is based on mathematical model involving decision tree and Markov model, early in the abstract. However, to further enhance clarity, we have modified the 'design' sub-heading on page 1 as:

"Design: Cost-utility analysis using mathematical model comprising of decision tree and Markov model was conducted to simulate direct costs and health outcomes over a lifetime horizon."

(2) There are potentially issues regarding multiple testing (12 screening strategies, 5 primary outcomes) inflating the risk of spurious results.

We thank the reviewer for the comment. We would like to clarify that we conducted a cost-utility analysis, which is a comparative analysis of costs and health outcomes in competing scenarios and is specifically designed to evaluate the cost-effectiveness of multiple interventions.[1] This standard methodology has been used to assess a range of interventions in India.[2,3] Within the scope of glaucoma as well, cost-utility analysis has been used to assess several competing scenarios and is considered a robust method to determine the cost-effective models of service delivery.[4]

---

[1] Drummond MF, Sculpher MJ, Claxton K, Stoddart GL, Torrance GW. *Methods for the Economic Evaluation of Health Care Programmes*. (4th ed.) Oxford University Press. 2015. https://books.google.co.uk/books?id=lvWACgAAQBAJ

[2] Kaur G, Chauhan AS, Prinja S, Teerawattananon Y, Muniyandi M, Rastogi A, Jyani G, Nagarajan K, Lakshmi P, Gupta A, Selvam JM, Bhansali A, Jain S. Cost-effectiveness of population-based screening for diabetes and hypertension in India: an economic modelling study. Lancet Public Health. 2022 Jan;7(1):e65-e73.

[3] Chauhan AS, Prinja S, Srinivasan R, Rai B, Malliga JS, Jyani G, Gupta N, Ghoshal S. Cost effectiveness of strategies for cervical cancer prevention in India. PLoS One. 2020 Sep 1;15(9):e0238291. doi: 10.1371/journal.pone.0238291. PMID: 32870941; PMCID: PMC7462298.

[4] Tuck MW, Crick RP. The cost-effectiveness of various modes of screening for primary open angle glaucoma. Ophthalmic Epidemiol. 1997;4(1):3–17.

The model-based economic evaluation allows for economic assessment of multiple interventions, which may be a limitation in randomized clinical trials. Therefore, the methods employed in this study are not likely to produce erroneous or unreliable outcomes.

Population characteristics such as sex and socioeconomic status are not considered, and the generalizability of the results should be discussed more.

We thank the reviewer for the comment. We would like to clarify that the hypothetical cohort used in the models reflects the age-specific characteristics of the Indian population. For input parameters that are likely to vary based on population characteristics (as outlined in Table S1 on page 2 of the supplementary material), we utilized age-specific prevalence, incidence, and mortality estimates from sample surveys representative of Indian population to populate the screening models. Most of the epidemiological parameters were derived from community-based studies conducted in India.

Additionally, the parameters related to care-seeking behaviour and out-of-pocket expenditures were obtained from the National Sample Survey, which collected data from 555,114 households across all districts of the country[5]. Cost parameters were sourced from the National Health Systems Cost Database, which includes estimates based on data collected from over 100 hospitals nationwide.[6] Therefore, the majority of the parameters in the model are derived from national-level surveys, ensuring the model is specific to the Indian context.

We specify that the population sub-groups were representative of age-specific socio-demographic distribution in India on page 5 (2nd paragraph) to ensure clarity for the readers.

Abstract

1. **Line** 30. It would be clearer to replace "considered" with "included" or similar.

We thank the reviewer for the comment. As suggested by the reviewer, we have replaced 'considered with 'included'. The rephrased sentence on page 1 is as follows:

Participants: A hypothetical cohort of different target population groups in terms of age groups and risk of glaucoma (age group 40-75 years, 50-75 years, 40-75 years age group at high risk of glaucoma, 50-75 years age group at high risk of glaucoma) were included in comparative screening strategies.

2. The "primary and secondary outcomes" subheading only lists primary outcomes, of which there are 5, multiplied by 12 screening strategies. If all of these are indeed primary outcomes, it sounds as if there are 60 primary comparisons? This significantly increases the risk of type 1

[5] Ministry of Statistics & Programme Implementation, Government of India. India - Household Social Consumption: Health, NSS 75th Round Schedule-25.0: July 2017-June 2018. Available from: http://microdata.gov.in/nada43/index.php/catalog/152, last accessed on 10.03.2025

[6] National Health System Cost Database from India. Available from: https://www.healtheconomics.pgisph.in/costing_web/index.php?action=gen_output, last accessed on 10.03.2025

error due to multiple testing, i.e. the risk of spurious results. The authors may wish to select fewer primary outcomes or apply statistical correction for multiple testing.

We thank the reviewer for the comment. We would like to clarify that the life years, QALYs, costs, and out-of-pocket expenditures for each screening scenario were compared with those of the usual care scenario to evaluate the net health gains and incremental costs, as presented in Table 1 of the main manuscript, in accordance with standard methodology.[7]

Furthermore, economic evaluations are used to compare costs and health outcomes across multiple scenarios using dominance and extended dominance analysis. However, in our analysis, none of the screening strategies were dominated, and as such, there was no need to perform extended dominance analysis.[7]

In response to the reviewer's suggestion, we have revised the outcomes section on page 1 to differentiate between primary and secondary outcomes as follows:

"Primary and Secondary Outcomes: The primary outcome was the incremental cost-utility ratio for each screening strategy in comparison to usual care. The secondary outcomes included per person lifetime costs, lifetime out-of-pocket expenditures, life years, and quality-adjusted life years (QALYs) across all screening scenarios and usual care."

3. Line 51-53. The results are quite vague – it would be more useful to state the precise screening programme. Point estimates should also include an uncertainty measure ,e.g. the 95% CI.

We thank the reviewer for the feedback. While we understand the reviewer's concern, due to word limit restrictions, it is not feasible to present the QALYs for all the screening scenarios in detail. However, to highlight the overall result, we have included a summary statement on page 1: "Depending on the type of screening strategy, the gain in QALY per person ranged from 0.006 to 0.046 relative to usual care." For the same reason of word count constraints, it may not be possible to provide the values at 95% confidence intervals.

The precise results are detailed on page 9 and 10 of the manuscript under the results section of the main manuscript.

Introduction

4. The introduction is clear and well written. However, it is not really explained why quality-adjusted life years seems to be the headline comparison (this is my assumption, based on this outcome being the first result reported in the abstract). What is the relevance of this measure, compared to say, risk of blindness?

We thank the reviewer for the comment. We would like to clarify that we conducted a cost-utility analysis, as stated in the second paragraph on page 4, in the introduction section. Cost-utility analysis typically compares QALYs and costs across different scenarios.[9] Since this is an integral part of the methodology, we further explain in the methods section that QALYs

---

[7] Drummond MF, Sculpher MJ, Claxton K, Stoddart GL, Torrance GW. *Methods for the Economic Evaluation of Health Care Programmes*. (4th ed.) Oxford University Press. 2015. https://books.google.co.uk/books?id=lvWACgAAQBAJ

were used in lieu of health outcomes for determining the incremental cost-utility ratios. Specifically, in the first paragraph on page 6, we state:

"Lifetime health outcomes were assessed in terms of per person gain in life years (LYs) and quality-adjusted life years (QALYs)."

Additionally, we would like to emphasize that the use of QALYs as the prime health outcome is consistent with the Indian guidelines for conducting health technology assessments.[8] The guidelines advocate for use of QALYs, so as to allow for comparison of health outcomes across interventions addressing different disease conditions. To ensure clarity for readers, we have referenced this as the second line in the methods section on page 4 (third paragraph):

"The choice of cost-utility analysis was guided by the Indian reference case for conducting economic evaluations."

5. The final paragraph states that the aim is to compare cost-utility – again I am unsure how that links to two of the primary outcomes: "life years, quality adjusted life years"

We thank the reviewer for the comment. We would like to clarify that we conducted a cost-utility analysis, as outlined in the second paragraph on page 4 of the introduction section. A cost-utility analysis typically compares QALYs and costs across different scenarios.[9] The use of QALYs as the primary health outcome is consistent with the Indian guidelines for conducting health technology assessments.[7]

Life years are utilized in the calculation of QALYs and are essential for analysing the impact of the intervention on life expectancy, as well as for validating the model and its findings. As a result, life years have been reported in the results section of the paper. In line with the reviewer's suggestion, we have listed life years (LYs) and QALYs as secondary outcomes in the abstract.

Methods

6. The beginning of the methods describes the development of the interventions, with 4 target populations x 2 screening methods x 2 screening frequencies. There are 16 possible combinations which result in 12 selected interventions. It would be helpful early in the methods to directly state (a list or table) which screening strategies were selected. It is also not clear which 4 possible combinations were dropped and why.

We thank the reviewer for the observation. In response to the reviewer's suggestion, we have revised the manuscript to provide clearer details about the screening scenarios in the introduction (second paragraph on page 4) as follows:

---

[8] Sharma D, Prinja S, Aggarwal AK, Rajsekar K, Bahuguna P. Development of the Indian Reference Case for undertaking economic evaluation for health technology assessment. Lancet Reg Health Southeast Asia. 2023 Jun 17;16:100241. doi: 10.1016/j.lansea.2023.100241. PMID: 37694178; PMCID: PMC10485782.

[9] Drummond MF, Sculpher MJ, Claxton K, Stoddart GL, Torrance GW. *Methods for the Economic Evaluation of Health Care Programmes*. (4th ed.) Oxford University Press. 2015. https://books.google.co.uk/books?id=lvWACgAAQBAJ

"Our study specifically examines the cost-utility of eight annual screening strategies based on different target population groups (age groups 40-75 years, 50-75 years, 40-75 years at high risk of glaucoma, 50-75 years at high risk of glaucoma), and screening methods [face-to-face screening (F2F) and AI-supported face-to-face screening (AI-based F2F)], relative to the usual care scenario. We also evaluated screening in the 40-75 years age group (universal versus risk-based screening) at a frequency of once every five years (Table 1)."

We would like to clarify that we did not evaluate once in five years' screening in the 50-75 years age group (e.g., F2F screening in high-risk populations aged 50-75 years, AI-based F2F screening in high-risk populations aged 50-75 years, F2F screening in the general population aged 50-75 years, and AI-based F2F screening in the general population aged 50-75 years) based on expert consultation.

7. It is only clear to me from page 5 line 37 that this is a simulation study and does not use patient data. This should be more clearly signposted in the abstract and introduction, because it is not directly stated.

We thank the reviewer for the comment. To clarify to the readers that this is a simulation study before the initiation of the methods section of the main manuscript, we have mentioned the study design in the 'design' sub-heading of the abstract on page 1 as:

"Cost-utility analysis using mathematical model comprising of decision tree and Markov model was conducted to simulate relevant costs and health outcomes over a lifetime horizon."

8. In light of the above I suggest reorganizing the methods. It is currently a bit muddled – the method to compare screening strategies is described before we know how the data was generated, or what the model includes. It will be easier to follow if it began by describing how the data was simulated – model parameters, outcomes, costs, and the 4 target populations. Followed by explaining how this was put together in a single model, including a list of all variables. Then describing the comparisons and statistical tests performed, and finally the sensitivity analyses.

We thank the reviewer for the comment. We would like to clarify that we have adhered to the standard format for reporting economic evaluations, in line with the published literature.[10,11,12] In the methodology for model-based economic evaluation, following the identification of interventions and comparators and outlining the key model characteristics such as perspective, time horizon, and discounting, the second step involves developing the

---

[10] Tang J, Liang Y, O'Neill C, Kee F, Jiang J, Congdon N. Cost-effectiveness and cost-utility of population-based glaucoma screening in China: a decision-analytic Markov model. Lancet Glob Health. 2019 Jul;7(7):e968-e978. doi: 10.1016/S2214-109X(19)30201-3. Epub 2019 May 20. PMID: 31122906.

[11] Ben ÂJ, Neyeloff JL, de Souza CF, Rosses APO, de Araujo AL, Szortika A, Locatelli F, de Carvalho G, Neumann CR. Cost-utility Analysis of Opportunistic and Systematic Diabetic Retinopathy Screening Strategies from the Perspective of the Brazilian Public Healthcare System. Appl Health Econ Health Policy. 2020 Feb;18(1):57-68. doi: 10.1007/s40258-019-00528-w. PMID: 31674001; PMCID: PMC6978298.

[12] Liu H, Li R, Zhang Y, Zhang K, Yusufu M, Liu Y, Mou D, Chen X, Tian J, Li H, Fan S, Tang J, Wang N. Economic evaluation of combined population-based screening for multiple blindness-causing eye diseases in China: a cost-effectiveness analysis. Lancet Glob Health. 2023 Mar;11(3):e456-e465. doi: 10.1016/S2214-109X(22)00554-X. Epub 2023 Jan 23. PMID: 36702141.

model structure. Based on this structure, the necessary parameters for populating the model are identified and sourced from secondary literature. Using these input parameters, we then calculate the lifetime costs and health benefits.[13]

We have reported these steps sequentially in the methodology to ensure clarity, as it is important for readers to understand the model structure before delving into the details of the model parameters.

However, to provide a concise overview of the methods used in our analysis, we present key details following the description of interventions and usual care in the 'Model Overview' section on pages 5 and 6.

The majority of the data used in the analysis were sourced from secondary literature (Table S1 of the supplementary material). The cost of screening was estimated normatively by identifying the resources required to implement screening at primary health centres, with the cost of these resources derived from the National Health System Cost Database for India, as detailed on page 7 under the sub-heading 'Cost of Screening and Treatment' and section S2 of the supplementary material.

9. Having read the methods several times, some important aspects are quite unclear to me.
a. how the base populations are generated. There are 4 target populations, differing by age and risk status, but it is hard to follow what parameters apply to each population and precisely how they were generated – I do not think I could replicate these populations given the current information.
b. The total list of variables included in the model. E.g, it is unclear whether aspects such as sex or socioeconomic status are considered – it is not stated so I assume not?
c. The total list of comparisons. From the abstract I assume there are 60, due to 12 interventions x 5 primary outcomes, but a concise explanation of the comparisons of interest is currently missing from the methods to confirm this.

We thank the reviewer for the comment. As correctly noted, our study considers four target populations, which vary by age and risk status. The parameters specific to each group, such as the prevalence of glaucoma at ages 40 and 50, age-specific incidence rates, and the risk of developing glaucoma in high-risk groups (e.g., those with hypertension, diabetes, or a family history of glaucoma), along with age-specific mortality rates, are detailed in Supplement Table S1. Due to word count constraints, the supplement contains comprehensive information on these input parameters.

The sources of these parameters include Indian sample survey studies, which are representative of the Indian population in terms of socio-demographic characteristics such as gender and socioeconomic status. This makes the analysis highly context-specific and directly applicable to the Indian population.

---

[13] Drummond MF, Sculpher MJ, Claxton K, Stoddart GL, Torrance GW. *Methods for the Economic Evaluation of Health Care Programmes*. (4th ed.) Oxford University Press. 2015. https://books.google.co.uk/books?id=lvWACgAAQBAJ

Additionally, the details of the screening costs, which were derived normatively, and the utility calculations for different target population groups, are also provided in the supplement (Section S2 and S3). This ensures transparent reporting and offers readers the necessary information to replicate this methodology in other regions globally.

We would like to specify that we used the standard method for cost-utility analysis and compared the interventions with usual care through computation of ICUR.[14] ICUR is the primary outcome and is calculated as ratio of net costs and net health gain (in terms of QALYs).

Results

10. It would be useful to begin the results by describing the simple characteristics of the 4 target populations.

We thank the reviewer for the comment. We would like to clarify that this is a model-based economic evaluation, where the target population represents the characteristics of Indian population, differing primarily by age and risk of developing glaucoma. These differences are determined by various screening strategies, as outlined in the methods section under the sub-heading 'Details of Screening Strategies and Target Population Groups' on pages 4 and 5.

The objective of the analysis is to evaluate the efficiency of different screening strategies and assess their impact on out-of-pocket expenditures. Given this focus, we believe that the characteristics of the four target populations are more appropriately presented in the methods section, rather than in the results section.

11. Table 1. The statistical method should be included in the table heading. Brackets are not explained. I don't think 4 decimal places is necessary and it harms the readability of this table, if reduced to 2dp it may be possible to fit all data on a single row. The cost columns are missing units (there is a symbol but not rendered). It is not clear why "40-70 years populations" are below the "50-70 years" subheading.

We thank the reviewer for the feedback. The values presented in the manuscript were computed using the Monte Carlo simulation method, and we have specified this in a footnote to Table 1.

We understand that 4 decimal points in the table may harm the readability of the table. However, given that the intervention affects the health outcomes at the fourth decimal point, therefore, it was deemed essential to report the values of health outcomes till fourth decision point for comparison between interventions.

Additionally, we have clarified the meaning of the brackets in the footnote: "Values in parentheses represent values at 95% confidence intervals."

[14] Drummond MF, Sculpher MJ, Claxton K, Stoddart GL, Torrance GW. *Methods for the Economic Evaluation of Health Care Programmes*. (4th ed.) Oxford University Press. 2015. https://books.google.co.uk/books?id=lvWACgAAQBAJ

The cost symbol '₹' is defined as Indian National Rupee in the footnote of Table 1, and this currency was consistently used for all cost-related figures, as outlined in the methods section on page 6 (first paragraph).

Furthermore, we would like to clarify that the first two sub-headings in Table 1 compare annual screening strategies in 40-75 years and 50-75 years old population, while the last sub-heading, "40-75 years populations," presents results for every five years' screening. Therefore, it is positioned below the "50-75 years population- annual screening" sub-heading to reflect this distinction in the screening frequency.

12. Table 2. Please add the statistical method. Are any uncertainty measures reported? I am unable to tell because it is not clear which method was used to create these percentages.

We thank the reviewer for the comment. We followed the standard methodology for performing probabilistic sensitivity analysis, as outlined in the 'sensitivity analysis' section of the methods on page 8 and 9.[15]

We specify the method in the footnote to the table 2 as- Probability of cost-effectiveness computed through probabilistic sensitivity analysis using Monte Carlo simulation method.

13. Throughout the paper various percentages are reported but again I am unsure regarding the certainty of these, and whether certainty estimates are generated, but if available they should be reported.

We thank the reviewer for the comment. In response, we conducted a scenario analysis to model the strengthening of public sector glaucoma care through public provisioning or publicly financed and strategically purchased care. A two-way sensitivity analysis was performed by varying the proportions of the population accessing public facilities and the differential availability of free medicines at these facilities. These details are outlined in the methods section on page 9, following standard methodological practices.[13]

The effect of change in percentages of public sector utilization and availability of free medicines on the incremental cost-utility ratios was measured. The values of percentages of public sector utilization and availability of free medicines, which were tested, were acquired through stakeholder consultation.

Discussion

14. A limitation which I did not see discussed is that the target populations seem to be treated as homogenous, but there could be variation due to sex, socioeconomic status, morbidity, which does not seem to be estimated here. So I wonder if this impacts the generalizability – who do these results apply to?

We thank the reviewer for the comment. We would like to clarify that the hypothetical cohort used in the models reflects the age-specific socio-demographic characteristics of the Indian

---

[15] Drummond MF, Sculpher MJ, Claxton K, Stoddart GL, Torrance GW. *Methods for the Economic Evaluation of Health Care Programmes*. (4th ed.) Oxford University Press. 2015. https://books.google.co.uk/books?id=lvWACgAAQBAJ

population. For input parameters that are likely to vary based on population characteristics (as outlined in Table S1 on page 2 of the supplementary material), we utilized age-specific prevalence, incidence, and mortality estimates from sample surveys representative of Indian population to populate the screening models. Most of the epidemiological parameters were derived from community-based studies conducted in India.

Additionally, the parameters related to care-seeking behaviour and out-of-pocket expenditures were obtained from the National Sample Survey, which collected data from 555,114 households across all districts of the country[16]. Cost parameters were sourced from the National Health Systems Cost Database, which includes estimates based on data collected from over 100 hospitals nationwide.[17] Therefore, the majority of the parameters in the model are derived from national-level surveys, ensuring the model is specific to the Indian context.

We specify that the population sub-groups were representative of age-specific socio-demographic distribution in India on page 5 (2nd paragraph) to ensure clarity for the readers. As such, the results are generalizable to the Indian population, which aligns with the primary aim of the study. Furthermore, the title of the study provides an early indication that the findings are applicable to the Indian population.

[16] Ministry of Statistics & Programme Implementation, Government of India. India - Household Social Consumption: Health, NSS 75th Round Schedule-25.0: July 2017-June 2018. Available from: http://microdata.gov.in/nada43/index.php/catalog/152, last accessed on 10.03.2025

[17] National Health System Cost Database from India. Available from: https://www.healtheconomics.pgisph.in/costing_web/index.php?action=gen_output, last accessed on 10.03.2025

Reviewer: 2
Dr. Olusola Olawoye, University College Hospital
Comments to the Author:
l congratulate the authors of this study. They have taken time to be as detailed and comprehensive as possible and the study provides information about the cost effectiveness of population screening for glaucoma in India. l have a few comments.

1. The authors mentioned in their methodology that “The current scenario is that of opportunistic diagnosis by the ophthalmologists at higher level of care. However what the authors are considering here is population screening for glaucoma. The diagnosis, can take place either at secondary or tertiary eye care. The authors seem to confuse screening with diagnosis. They seem to compare population-based glaucoma screening strategies with opportunistic glaucoma diagnosis, which is performed by ophthalmologists. Page 5 lines 5-7.

We thank the reviewer for the comment. As rightly noted by the reviewer, we compare population-based glaucoma screening strategies with opportunistic glaucoma diagnosis, which is performed by ophthalmologists, which is the current practice in India. The intervention scenario is population-based screening for glaucoma followed by diagnosis and treatment at secondary or tertiary care. The counterfactual scenario is the current practice-opportunistic diagnosis and treatment by the ophthalmologists at higher level of care in absence of the systematic universal screening.

In accordance with the Indian reference case for conducting economic evaluation, we compared the screening scenarios with the usual care.[18]

2. The authors compared opportunistic diagnosis with population screening. It would seem that both might not be cost-effective. Did the authors evaluate whether opportunistic diagnosis is cost-effective? Comparing these two (population screening and opportunistic diagnosis) would seem to mean that opportunistic diagnosis is cost-effective.

We thank the reviewer for the comment. As indicated implicitly from the analysis, opportunistic diagnosis for glaucoma is cost-effective when compared to a population-level screening programme.

We did not compare the opportunistic diagnosis and population screening with ‘do nothing’ scenario, in accordance with the Indian reference case for conducting economic evaluation. Additionally, comparing with a ‘do nothing’ scenario raises ethical concerns. If interventions were found to be not cost-effective relative to a ‘do nothing’ scenario, it would be unethical to cease providing treatment to diagnosed cases.

---

[18] Sharma D, Prinja S, Aggarwal AK, Rajsekar K, Bahuguna P. Development of the Indian Reference Case for undertaking economic evaluation for health technology assessment. Lancet Reg Health Southeast Asia. 2023 Jun 17;16:100241. doi: 10.1016/j.lansea.2023.100241. PMID: 37694178; PMCID: PMC10485782.

3. The authors said that their study was similar to studies in high-income countries; however these studies are entirely different as the study by Burr et al compared population screening with no screening at all, but this study is comparing population screening with opportunistic diagnosis at tertiary level of care

We thank the reviewer for the comment. We agree that the results of our study are consistent with those of studies conducted in high-income countries. While there may be differences in study parameters of a high-income country, when compared to a country, as India, which has a higher prevalence of glaucoma and lower healthcare costs, screening interventions remain to be cost-ineffective. We have specified the same on page number 12 (second paragraph) as:

"Our study's results are consistent with those observed in numerous high-income countries, which indicate that the screening is likely to be cost-ineffective in low-and-middle income countries, despite the higher prevalence of glaucoma and lower healthcare costs"

As the reviewer noted, Burr et al. compared population screening with no screening at all. However, in their analysis, the 'no screening' scenario also implied opportunistic detection and treatment at higher levels of care, which is analogous to our approach.

4. The authors referred to a study in the United States where opportunistic diagnosis of glaucoma during routine ophthalmologic examinations is considered cost-effective relative to scenarios involving no diagnosis or treatment. This baseline is fundamental as it gives a yardstick to understand the comparison. The authors might have done this first before comparing opportunistic diagnosis to population study

We thank the reviewer for the comment. In our study, we compared population-based glaucoma screening strategies followed by diagnosis and treatment at higher level of care with opportunistic glaucoma diagnosis and treatment, which is currently performed by ophthalmologists in India. According to the Indian reference case for conducting economic evaluations, interventions should be compared with the current practice (usual care). Therefore, in accordance with these guidelines and after consulting with experts, we did not compare the screening strategies with a 'do nothing' scenario.

5. Some of the references are wrong. The authors need to go over all the references. The references are not correct. Kindly check references 8 and 9 which the authors said were studies done in Asia. The current references there do not match some of the statements

We thank the reviewer for the comment. References 8 and 9 are studies conducted in China and India, both of which represent Asian populations. In response to the reviewer's suggestion, we have carefully re-checked all the references cited in the study.

6. The authors did not mention anything about health insurance. It would be interesting to know how this affects the cost-effectiveness of screening and out-of-pocket payments.

We thank the reviewer for the comment. We agree that health insurance can influence the cost-effectiveness of screening and out-of-pocket payments. However, the current government health insurance schemes in India primarily focus on inpatient care, while most glaucoma cases are managed in outpatient ophthalmic departments, which is out of the purview of the insurance schemes. Therefore, although we recommend expanding the essential medicines list to include common anti-glaucoma medications provided at government-owned or funded institutions at no or subsidized cost, we chose not to emphasize the role of health insurance in our analysis.

As the reviewer rightly pointed out, insurance may play a significant role in countries where the health system is based on insurance-led models. However, we believe that in the Indian context, health insurance will not have a substantial impact on glaucoma care until it is extended to include outpatient services.

7. The authors mentioned that patients with hypertension and diabetes are at high risk for glaucoma using some studies conducted in India. These medical diseases in themselves potentially increase the rate of blindness from glaucoma in patients with them. It is hoped that the authors did not factor in the treatment of these other co-existing diseases

We thank the reviewer for the comment. As noted by the reviewer, we would like to clarify that the analysis did not include the treatment costs for co-existing conditions such as diabetes and hypertension. The analysis exclusively considered the costs associated with screening, diagnosis, and treatment of glaucoma.

8. The authors may consider rephrasing their conclusions to sort of reflect this: Population screening using the current glaucoma care pathways, where most persons are seen in private facilities in India, is not cost-effective. To make it cost-effective, the government must provide some financing for diagnosis and treatment, and medications must be available.

We thank the reviewer for the valuable comment. As suggested by the reviewer, we have revised the conclusion on page number 15 (last paragraph) as:

"Although the Indian government is exploring the expansion of eye care services within a comprehensive primary healthcare framework, implementing a glaucoma screening program may not be cost-effective in the current context of care seeking behaviours skewed towards private sector. The study highlights the need to prioritize enhancing continuity of care by fortifying connections between primary healthcare and hospitals. Strengthening PHC oriented health systems through adequate financing for diagnosis and treatment within the government funded institutions is expected to not only improve the efficiency of glaucoma screening programs but also reduce the overall cost of glaucoma care at societal level."

VERSION 2 - REVIEW

**Reviewer**	**1**
**Name**	**Dack, Kyle**
**Affiliation**	**University of Bristol, MRC Integrative Epidemiology Unit**
**Date**	**16-Mar-2025**
**COI**	

The authors have made significant improvements to the paper, improving the clarity and accessibility. There were a number of statistical concerns in my original review which the authors either made changes to address, or gave reasonable explanations of in the response letter.

Title: I think the title should clarify that this uses simulated data, as this is a core part of the "setting". Perhaps "simulated population-based".

Table 1. I am unsure if it is only a local issue for me, but in my pdf copies the cost symbol in brackets (₹) did not render.
